# Flourishing in Early Adulthood Among Victimized Children: A Longitudinal Cohort Study

**DOI:** 10.1016/j.jadohealth.2026.02.010

**Published:** 2026-03-14

**Authors:** Flora Blangis, Louise Arseneault, Andrea Danese, Rachel M. Latham, Terrie E. Moffitt, Stefan Sprinckmoller, Yichang Wang, J. Kathy Xie, Helen L. Fisher

**Affiliations:** a Social, Genetic & Developmental Psychiatry Centre, Institute of Psychiatry, Psychology & Neuroscience, King’s College London, London, United Kingdom; b Department of Child and Adolescent Psychiatry, Institute of Psychiatry, Psychology & Neuroscience, King’s College London, London, United Kingdom; c National and Specialist CAMHS Clinic for Trauma, Anxiety, and Depression, South London and Maudsley NHS Foundation Trust, London, United Kingdom; d ESRC Centre for Society and Mental Health, King’s College London, London, United Kingdom; e Department of Psychology and Neuroscience, Duke University, Durham, North Carolina; f Department of Psychiatry and Behavioral Sciences, Duke University, Durham, North Carolina; g Duke University Population Research Institute, Duke University, Durham, North Carolina; h Department of Psychology, PROMENTA, University of Oslo, Oslo, Norway

**Keywords:** Adversity, Child abuse, E-Risk cohort study, Resilience, Thriving, Young adults

## Abstract

**Purpose:**

Childhood victimization has lifelong adverse consequences including lower well-being and functional impairments in adulthood. Nonetheless, some victimized individuals appear to fare well, although it remains unclear whether they flourish (i.e., experience overall well-being and optimal functioning).

**Methods:**

We used data from the nationally representative Environmental Risk (E-Risk) Longitudinal Twin Study, which followed 2,232 children born in 1994–1995 across England and Wales to 18 years of age (93% retention, N = 2,066). Victimization was prospectively assessed between ages 5–12 years, capturing exposure to severe physical abuse, sexual abuse, emotional abuse and neglect, physical neglect, peer bullying, and domestic violence. Flourishing was assessed at age 18 across four domains: social well-being, education and cognition, physical health, and mental well-being.

**Results:**

Over a third of the 558 victimized children flourished on at least one measure within all four domains at age 18, with the highest flourishing rates found for perceptions of social support, social status, and sleep quality. Experiencing two or more types of victimization was associated with lower flourishing for perceived social status (adjusted odds ratio [adj.OR] = 0.45, 95% confidence interval [CI] = 0.28, 0.73, *p* = .001) and faster biological aging (adj.OR = 0.57, 95% CI = 0.35, 0.93, *p* = .024) compared to single victimization exposure. Victimized girls were more likely to flourish in their educational attainment than boys (adj.OR = 1.61, 95% CI = 1.02, 2.55, *p* = .042).

**Discussion:**

A substantial proportion of victimized children flourished across multiple domains in early adulthood. Identifying enabling factors could inform promotive interventions.

Childhood victimization, which includes physical, sexual, and emotional abuse, neglect, exposure to domestic violence, and bullying by peers, has lifelong adverse effects including functional impairments in adulthood [1,2]. The consequences of childhood victimization affect multiple domains: social and behavioral functioning (e.g., social connections, involvement in crime, and violence) [3], economic outcomes (e.g., education and employment) [1], health-related behaviors (e.g., smoking and alcohol consumption) [4], and psychological well-being (e.g., life satisfaction) [5]. This is particularly concerning in early adulthood because outcomes during this developmental stage have a major impact on individuals’ health, well-being, and prosperity throughout adult life [6]. Nonetheless, some children appear to fare well after experiencing victimization [7]. However, it is unclear if they exceed expectations and achieve a state of flourishing, defined as a multidimensional concept of overall well-being and optimal functioning—not solely the absence of pathology [2,8,9].

Flourishing has been conceptualized in various ways in the literature but usually encompasses multiple domains, including feelings of happiness and life satisfaction [10], social well-being including having supportive and rewarding relationships [10], education, skills, and cognitive ability [11], and mental and physical health [9,10]. Together, these domains capture distinct aspects of flourishing and move beyond resilience to mental and physical illness toward focusing on the capacity to prosper [8].

Existing knowledge on flourishing in early adulthood after childhood victimization is limited. Most studies investigating functioning in adults who experienced victimization during childhood reported poorer outcomes compared to non-victimized individuals [2,12]. However, these studies did not examine whether a subset of victimized individuals could flourish across multiple domains despite their adverse experiences. Moreover, previous research often focused on single domains of functioning, such as psychosocial well-being [13,14] or socioeconomic outcomes [12], without considering whether those who have been victimized can flourish across multiple domains. Furthermore, existing research has focused on single, specific types of childhood victimization [13], which could be misleading because children often experience more than one type of victimization [15]. The strength of associations between victimization and functional outcomes also varies depending on whether prospective or retrospective measures of victimization are used [16], possibly because these different measures result in largely nonoverlapping groups of children being classified as exposed to victimization [17]. Many existing studies have also relied on cross-sectional designs and thus cannot establish the temporal order of victimization and flourishing, which limits the ability to establish causality [2,12–14,18]. In addition, examining potential sex differences in flourishing across multiple domains after childhood victimization is needed. Epidemiological and neurobiological studies suggest that women are more likely to flourish than men in early adulthood [19], but whether this occurs following exposure to different types of victimization and across multiple domains remains unclear.

To address these limitations, we used prospectively collected data from the Environmental Risk (E-Risk) Longitudinal Twin Study, a large, nationally representative cohort of children born in the United Kingdom. This cohort’s prospective longitudinal design, with functioning outcomes measured after comprehensive, multi-informant assessments of childhood victimization, overcomes the limitations of cross-sectional studies and enables investigation of associations between exposure to various individual and multiple types of victimization (polyvictimization), and functional outcomes across multiple domains. Some victimization types were also assessed retrospectively, facilitating exploration of whether rates of flourishing are similar (or different) for prospectively and retrospectively measured exposure to childhood victimization. Moreover, the sample also comprises roughly equal numbers of men and women, enabling examination of potential sex differences.

Therefore, we aimed to investigate whether some individuals exposed to any type of victimization in childhood can flourish when they reach adulthood and whether flourishing occurs across multiple domains of functioning or only in certain ones. In addition, we aimed to determine whether flourishing varies by type of victimization experienced, by prospective versus retrospective measures of victimization, by exposure to single versus multiple types of victimization, and/or by biological sex.

## Methods

### Study cohort

Participants were members of the E-Risk Longitudinal Twin Study, which tracks the development of a nationally representative birth cohort of 2,232 twin children born in England and Wales in 1994–1995. Full details about the sample are reported elsewhere [20] and in Text S1. Briefly, the E-Risk sample was constructed in 1999–2000 when 1,116 families (93% of those eligible) with same-sex 5-year-old twins (of whom 90.4% were White) participated in home-visit assessments. This sample comprised 56% monozygotic and 44% dizygotic twin pairs; sex was evenly distributed within zygosity (49% male). Families were recruited to represent the UK population of families with newborns in the 1990s, on the basis of residential location throughout England and Wales and mother’s age. E-Risk families are representative of UK households according to the Index of Multiple Deprivation (online Text S1 and Figure S1).

Follow-up home-visits were conducted when children were aged 7, 10, 12, and 18 years (participation rates were 98%, 96%, 96%, and 93%, respectively). At age 18, 2,066 participants were assessed (47% male). There were no differences between those who did and did not take part at age 18 in terms of socioeconomic status (SES) assessed when the cohort was initially defined (χ^2^ = 0.86, *p* = .65), age-5 intelligence quotient (IQ) scores (*t* = 0.98, *p* = .33), age-5 internalizing or externalizing behavior problems (*t* = 0.40, *p* = .69 and *t* = 0.41, *p* = .68, respectively), or childhood polyvictimization (*z* = 0.51, *p* = .61).

The Joint South London and Maudsley and Institute of Psychiatry Research Ethics Committee approved each study phase (NRES 1997/122). Parents gave informed consent and twins gave assent between 5 and 12 years and then informed consent at age 18. This study followed the Strengthening the Reporting of Observational Studies in Epidemiology (STROBE) guidelines (Table S1).

### Measures

#### Childhood victimization.

Prospective measures of victimization utilized in this cohort and the coding criteria are described elsewhere [21] and in Text S2. In brief, lifetime exposure to several types of victimization was assessed repeatedly when children were 5, 7, 10, and 12 years. Comprehensive dossiers were compiled for each child with cumulative information about the following: exposure to domestic violence between mother and partner; frequent bullying by peers; physical abuse by an adult; sexual abuse; emotional abuse and neglect; and physical neglect, between birth and age 12. Dossiers comprised reports from caregivers, recorded narratives of caregiver interviews, recorded debriefings with research workers who had coded any indications of abuse and neglect at any of the successive home visits, interviews with children about their bullying experiences, and information from clinicians whenever the study team made a child-protection referral. These were reviewed by two independent researchers and rated for the presence and severity (none/mild/severe) of each type of victimization (Text S2). Exposure to any type of victimization was defined as experiencing severe physical abuse, sexual abuse, emotional abuse and neglect, physical neglect, exposure to domestic violence, or bullying by peers.

We also used victimization measured retrospectively using the Childhood Trauma Questionnaire (CTQ) (Text S2) [22]. At age 18, participants reported on their personal experiences of physical, sexual, and emotional abuse, and physical and emotional neglect, for the period before they were aged 12 [17]. Retrospective exposure to any victimization was defined as experiencing any type of moderate or severe victimization on the Childhood Trauma Questionnaire or prospectively reported domestic violence or bullying by peers to ensure all comparable types were captured.

#### Early adulthood flourishing.

Drawing on the various conceptual frameworks of flourishing identified above and considering both the age of participants (early adulthood) and the variables available in our study, we explored functional outcomes at age 18 across four conceptually distinct domains including 15 different measures: (1) social well-being (i.e., high perception of social status and high perception of social support), (2) education and cognition (i.e., high educational attainment, high perceived ability to get ahead in life, and high cognitive functioning), (3) physical health (i.e., high levels of physical activity, good sleep quality, and slower biological aging), and (4) mental well-being (i.e., high life satisfaction) (more details in Table 1 and Text S3). Because victimized children may flourish in some areas of functioning but not in others, we analyzed each measure separately to best capture their potentially heterogeneous outcomes.

#### Confounders.

Individual-level covariates included biological sex at birth and IQ at age 5. Family-level covariates included family SES at age 5 and family psychiatric history assessed at age 12. Details on these covariates are provided in Text S4.

### Statistical analyses

First, we tested in the whole sample the associations between exposure to any type of severe victimization between birth and age 12 and the measures within each domain of functioning at age 18. We conducted linear regression analyses for continuous variables and binary logistic regression analyses for categorical variables, subsequently adjusting for biological sex, age-5 IQ, family SES, and family psychiatric history. These initial analyses confirmed whether exposure to any type of victimization by age 12 was associated with poorer functional outcomes.

Second, in the subsample of children exposed to any type of severe victimization, we investigated whether some victimized children flourish at age 18 by ascertaining the percentage performing better than other victimized peers across functional measures identified as significant in Step 1 (*p* < .05). Consistent with previous analyses in this cohort [23], for continuous variables we used the standardized residuals obtained from the multiple regression analyses conducted in Step 1, which indicate the difference between actual and predicted scores based on victimization exposure (Figure S2). Victimized children with residuals >0 (their actual score was greater than their predicted score) were classified as “flourishing”, while those scoring 0 (no difference between actual and predicted score) or <0 (actual score was worse than predicted score) were classified as “not flourishing”. For the only categorical outcome, educational attainment, children who achieved one or more A levels were classified as flourishing (best possible outcome at this age), while those with no qualifications or only secondary school qualifications (GCSEs) were classified as not flourishing. We repeated these analyses using retrospectively assessed victimization to determine whether similar findings were observed.

Among the subsample of victimized individuals with prospectively assessed victimization, we next conducted binary logistic regression analyses to explore the association with flourishing for (1) each type of victimization (e.g., physically abused vs. other types of victimization), (2) polyvictimization (no = only exposed to one type of victimization, yes = exposure to two or more types), and (3) biological sex (Figure S3).

As a sensitivity analysis, we defined flourishing as a continuous variable using standardized residuals >0. We conducted linear regression analyses to explore the association with flourishing for (1) each type of victimization (e.g., physically abused vs. other types of victimization), (2) polyvictimization (no = only exposed to one type of victimization, yes = exposure to two or more types), and (3) biological sex. Finally, we repeated all these analyses using an enhanced definition of flourishing–namely victimized individuals with standardized residuals in the top 25th percentile (consistent with previous analyses in this cohort) [23]. These analyses were only conducted for associations that were statistically significant (at *p* < .05) in the main analyses where flourishing was defined more broadly.

All analyses accounted for the nonindependence of twin observations using the cluster command within STATA 16.1 (StataCorp, College Station, TX, USA). The premise and analysis plan for this project were preregistered at https://sites.duke.edu/moffittcaspiprojects/files/2024/11/Blangis_ERisk_Flourishing-among-victimized-kids_final_20NOV2024.pdf.

## Results

### Is exposure to victimization during childhood associated with poorer functional outcomes at age 18?

Among the 2,066 participants included in the analyses, 558 (27%) were exposed to any type of severe victimization (49% male) (see Table 2 for descriptive statistics for the overall sample and by victimization status). Compared to individuals not exposed to severe victimization, individuals exposed to victimization had significantly lower perceptions of their social status, social support, and their ability to get ahead in life, poorer performance in spatial span reversed tasks, longer spatial working memory mean time, lower sleep quality, faster biological aging, lower life satisfaction, and less educational attainment after adjustment for confounders (Table 3). Similar results were obtained when using retrospective measures of victimization (Table S2).

### Are some individuals exposed to victimization in childhood able to flourish when they reach adulthood?

**Figure 1.**
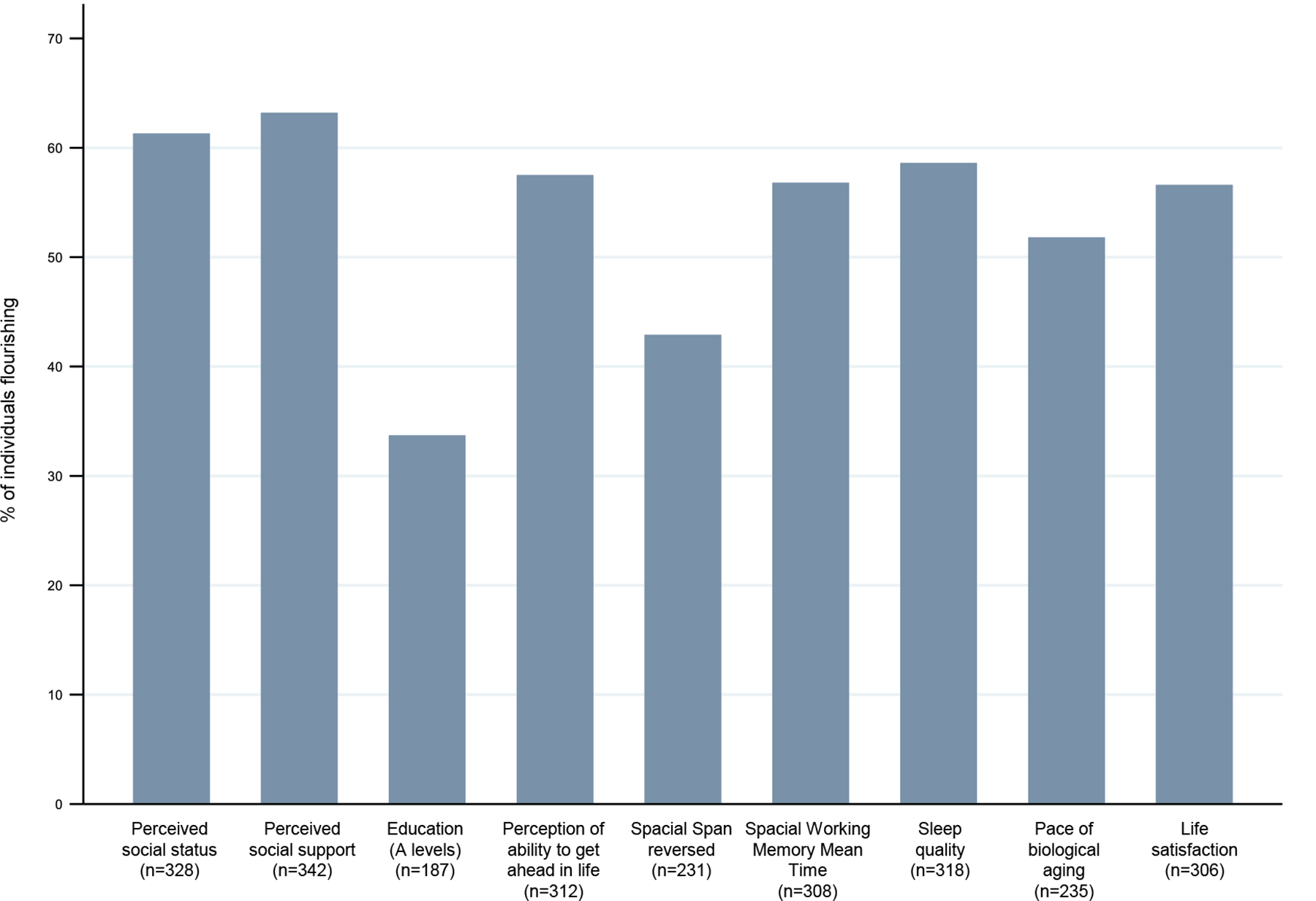
Proportion of individuals who are flourishing in early adulthood after exposure to any type of severe victimization in childhood. (For continuous outcome variables, we used the standardized residuals obtained from the multiple regression analyses, which indicate the difference between actual and predicted scores on each outcome variable based on any severe victimization exposure between 0–12 years of age. Victimized children with residuals >0 (their actual score was greater than their predicted score) were classified as “flourishing” on that outcome measure, while those scoring 0 (no difference between actual and predicted score) or <0 (actual score was worse than predicted score) were classified as “not flourishing” on that outcome measure. For the only categorical outcome, educational attainment, children who achieved one or more A levels were classified as flourishing (best possible outcome at this age), while those with no qualifications or only secondary school qualifications (GCSEs) were classified as not flourishing.).

Between 34% and 63% of the 558 victimized children were found to be flourishing at age 18 on individual measures of functioning (Figure 1), with the highest rates for perceived social support (63%), perceived social status (61%), and sleep quality (59%). Approximately 55% of victimized children were flourishing on 5 or more of the 9 individual measures (Table S3) and 39% (n = 216) were flourishing on at least one measure within all four domains. Similar results were obtained when using retrospective measures of victimization (Table S4) and thus retrospectively reported victimization was not explored in subsequent analyses.

### Does flourishing vary by type of victimization experienced?

Individuals exposed to bullying by peers were significantly less likely to flourish in relation to perceived social support (adjusted odds ratio (adj.OR) = 0.59; 95% confidence interval (CI) = 0.39, 0.90; *p* = .015) and perceived ability to get ahead in life (adj.OR = 0.66; 95% CI = 0.44, 0.98; *p* = .037), and were significantly more likely to flourish in spatial span reversed performance (adj.OR = 1.61; 95% CI = 1.10, 2.35; *p* = .014), compared to individuals exposed to other types of victimization (Table S5). Individuals exposed to physical abuse were less likely to flourish in perceived social support and status (adj.OR = 0.61; 95% CI = 0.38, 0.98; *p* = .043 and adj.OR = 0.45; 95% CI = 0.26, 0.78; *p* = .004, respectively) compared to individuals exposed to other types of victimization. Those exposed to emotional abuse and neglect were significantly less likely to flourish in perceived social status (adj.OR = 0.34; 95% CI = 0.17, 0.70; *p* = .003), and individuals exposed to sexual abuse were significantly less likely to flourish in spatial span reversed performance (adj.OR = 0.32; 95% CI = 0.11, 0.94; *p* = .039) and life satisfaction (adj.OR = 0.26; 95% CI = 0.08, 0.81; *p* = .020), compared to individuals exposed to other types of victimization.

### Does flourishing vary by exposure to single versus multiple severe types of victimization?

Flourishing varied by amount of victimization exposure; polyvictimization was significantly associated with lower flourishing for perceived social status (adj.OR = 0.45; 95% CI = 0.28, 0.73; *p* = .001), perceived ability to get ahead in life (adj.OR = 0.64; 95% CI = 0.41, 0.99; *p* = .044), pace of biological aging (adj.OR = 0.57; 95% CI = 0.35, 0.93; *p* = .024), and life satisfaction (adj.OR = 0.61; 95% CI = 0.38, 0.98; *p* = .043), compared to single victimization exposure (Table S6).

### Does flourishing vary by biological sex?

Victimized girls were significantly more likely to flourish in educational attainment than boys (adj.OR = 1.61; 95% CI = 1.02, 2.55; *p* = .042) but no other sex differences were found (Table S7).

### Sensitivity analyses

Using a continuous definition of flourishing, individuals exposed to domestic violence had lower levels of flourishing in perceived social support (adj.*ß* = −0.08, 95% CI = −0.15, −0.00, *p* = .042) but higher levels of flourishing in life satisfaction (adj.β = 0.27 95% CI = 0.12, 0.41, *p* < .001), compared to individuals exposed to other forms of victimization (Table S8). Individuals exposed to physical abuse had lower levels of flourishing in sleep quality (adj. *ß* = −0.20 95% CI = −0.34, −0.05, *p* = .008) and life satisfaction (adj. *ß* = −0.24, 95% CI = −0.44, −0.04, *p* = .019), compared to those exposed to other forms of victimization. Individuals exposed to sexual abuse had lower levels of flourishing in sleep quality (adj. *ß* = −0.31 95% CI = −0.61, −0.01, *p* = .040) and individuals exposed to physical neglect had lower levels of flourishing in biological aging (adj. *ß* = − 0.43 95% CI = −0.63, −0.24, *p* < .001), compared to those exposed to other forms of victimization. Levels of flourishing did not vary between those exposed to single versus multiple types of victimization (Table S9). Girls showed lower levels of flourishing in perceived social support than boys (adj. *ß* = −0.11 95% CI = −0.18, −0.04, *p* < .002) (Table S10).

Using an enhanced definition of flourishing, the proportion of individuals flourishing was lower across all domains of functioning (Table S11). Individuals exposed to bullying were significantly less likely to flourish in perceived social support compared to individuals exposed to other forms of victimization (adj.OR = 0.54; 95% CI = 0.36, 0.82; *p* = .004) (Table S12). Polyvictimized individuals showed significantly accelerated biological aging compared to those exposed to single victimization (adj.OR = 0.55; 95% CI = 0.34, 0.90; *p* = .017) (Table S13).

## Discussion

In this study, we investigated whether some individuals exposed to any severe victimization in childhood were able to flourish when they reached adulthood, exploring a relatively new concept compared to the more traditional focus on the absence of pathology. Victimized children did worse than non-victimized children for many of the functional measures investigated, with the exception of some aspects of cognitive functioning, regardless of whether we measured victimization prospectively or retrospectively. While victimization during childhood is inherently harmful and should always be prevented, our results suggest that some individuals can flourish. Between approximately one-third to two-thirds of the victimized children included in our study flourished at age 18, and flourishing occurred across all four domains for around 40% of victimized individuals. The highest rates of flourishing were evident for social well-being (i.e., perceived social status and social support) and physical health (i.e., slower biological aging and better sleep quality). Flourishing also varied according to the type and amount of victimization and biological sex.

Consistent with previous literature, victimized children tended to do worse across functional outcomes than non-victimized children [1,3,5]. However, we did not find a significant association between childhood victimization and most cognitive function tests, likely due to the adjustment for age-5 IQ, which attenuates the effect of childhood victimization on cognition [21]. Moreover, we found that many victimized individuals flourished when they reached adulthood, across multiple functional domains. This extends limited prior research on whether some victimized children can flourish rather than just avoid negative outcomes. For instance, a large cross-sectional Canadian study found that just over half of adults who reported childhood abuse (physical or sexual abuse, intimate partner violence) had high levels of positive functioning and emotional well-being [24].

In our sample, individuals exposed to bullying by peers, physical abuse, emotional abuse and neglect were less likely to flourish in the social well-being domain compared to those exposed to other types of victimization. This aligns with previous research showing lower social well-being in adulthood among individuals physically or emotionally abused in childhood [25], as well as fewer social relationships in early adulthood following peer victimization [26]. Individuals exposed to bullying by peers who were classified as flourishing showed better performance on the spatial span reversed task, involving visuospatial working memory capacity. This may indicate an adaptive cognitive response to social adversity, such as increased vigilance. However, childhood bullying has been associated with poorer cognitive functioning in adulthood in previous research [27], so our finding requires further exploration. In our study, children exposed to sexual abuse were less likely to report life satisfaction in early adulthood, which is consistent with previous studies of adults sexually abused in childhood [28]. As expected, children who experienced polyvictimization—a group already known to be at higher risk for adverse outcomes [15]—were less likely to flourish than those who experienced single victimization. Our hypothesis that girls are more likely to flourish was only supported in terms of educational attainment, with victimized girls outperforming victimized boys, a gap already well-documented among the general population [29]. Sensitivity analyses using a continuous definition of flourishing provided greater granularity regarding the level of flourishing among victimized individuals, particularly across different types of victimization. Sensitivity analyses using an enhanced definition of flourishing unsurprisingly resulted in a lower proportion of victimized individuals classified as flourishing. Nonetheless, we found similar effect sizes for associations, albeit these were not statistically significant likely due to the reduced number of individuals in this group.

### Strengths and limitations

Our study has several strengths. First, we used a nationally representative cohort study with a low attrition rate at age 18, ensuring that individuals with poorer outcomes were not overrepresented among those lost to follow-up. Therefore, the sample is not biased towards those likely to flourish. Second, the longitudinal design allowed for the collection of prospective measures of victimization and functional outcomes, providing reassurance that victimization occurred prior to assessment of flourishing. Third, we used extensive multi-informant victimization measures supplemented by retrospective reports, ensuring a comprehensive assessment and enabling testing of different approaches to classifying victimization exposure. Fourth, we examined both individual types of victimization and the impact of polyvictimization, as well as potential differences by biological sex. Finally, unlike many previous studies that focused on a single domain [12–14], we assessed multiple aspects of flourishing, offering a more holistic perspective on well-being.

Our study also has several limitations. First, our main analyses focused on any type of severe victimization rather than investigating each type individually due to sample size constraints. In addition, in secondary analyses, we compared each type of victimization to all others rather than exploring every possible combination, as we lacked statistical power. Second, we selected functional outcomes based on the limited existing literature on this topic and the variables available in our study. Other factors, such as positive interpersonal relationships, community engagement, meaning in life, as well as positive affect (e.g., happiness) or gratitude [14,18], might have provided additional insights. Third, while our definition of flourishing was based on prior research using the E-Risk study, no standardized definition exists. Due to the structure of the available data, we were not able to use existing flourishing scales [8,9,40] and therefore could not sum our items into a composite score. Sensitivity analyses using a more restrictive definition resulted in fewer individuals classified as flourishing, highlighting the need for consensus. Moreover, the definition of flourishing may also vary across cultural contexts. Fourth, we assessed flourishing at a single time point, yet it is likely a dynamic concept that evolves across the life course, highlighting the need for longer follow-up studies [30]. Fifth, our results are derived from a cohort of predominantly White participants (comparable to the population of England and Wales in the 1990s; https://www.ons.gov.uk/peoplepopulationandcommunity/culturalidentity/ethnicity/articles/ethnicityandnationalidentityinenglandandwales/2012-12-11#changing-picture-of-ethnicity-over-time) and thus cannot be generalized to young people from other ethnic groups who may experience victimization and flourishing differently. Nonetheless, the E-Risk sample is representative of UK families in terms of geographical and socioeconomic distribution [1,20,23]. Future research should allow for the analysis of potential moderators, including race, ethnicity, and SES. Finally, this sample consisted exclusively of twins, which may limit generalizability. Although previous research suggests that the experience of victimization is comparable between twin and non-twin populations [15], shared environmental influences such as family context or social support may allow twins to flourish more than singletons. This highlights the need for replication in cohorts of singletons.

### Implications

Our findings suggest that despite the challenges associated with childhood victimization, some individuals can flourish in early adulthood. However, it is important to acknowledge that victimized children continue to experience significantly worse outcomes than their nonvictimized peers, and not all of them flourish [1,3,5]. Efforts should focus on creating environments and support systems that facilitate flourishing. Personalized interventions should be developed to promote flourishing based on the specific type of victimization experienced. For example, children exposed to bullying may benefit from interventions aimed at improving social well-being, such as peer support programs. One such program, the KiVa antibullying program, has been effective in reducing bullying victimization but has not been shown to improve well-being among victimized children [31]. This highlights the need to further explore the factors that enable flourishing to develop targeted interventions. For example, some personal capacities such as positive self-worth might influence a child’s perception of the world, while social support might provide additional resources to navigate challenges. In addition, our results indicate that boys who have been victimized may require additional support to achieve better educational attainment. Previous research has also shown that boys who have experienced bullying are more likely to be absent from school [32]. Interventions designed to improve academic outcomes for victimized boys, notably those promoting school engagement, could help mitigate the long-term educational disparities associated with victimization. Finally, in order to scale up these interventions, they could be implemented in schools, health care systems (e.g., the National Health Service (NHS) in the United Kingdom), or social care services. Further research is needed to identify the most appropriate interventions and to optimize their safety and effectiveness.

### Conclusions

Although victimized children generally did worse than their nonvictimized peers, a substantial proportion flourished across multiple domains when they reached early adulthood. However, children exposed to certain types of victimization or polyvictimization were less likely to flourish. These findings call for investigation of the factors that enable victimized children to achieve more than might be expected to inform promotive interventions. In addition, further research is needed to establish a standardized definition of flourishing to ensure consistency and comparability across studies. Longitudinal studies are also essential to understand the trajectory of flourishing and to identify critical periods for intervention.

## Supplementary Material

1

Supplementary Data

Supplementary data related to this article can be found at https://doi.org/10.1016/j.jadohealth.2026.02.010.

## Figures and Tables

**Table 1:** Measures of functional outcomes and definition of flourishing

Measure	Description of the measure	Definition of flourishing
Social well-being		
Perceived social status	Perceived family social status was measured at age 18 using an adapted version of the MacArthur SES measure [33]. Participants were shown an image of a ladder with five rungs and told that “this ladder represents how things are in the United Kingdom. At the top of the ladder are all the people who have the best jobs, lots of money, live in nice places, and go to the best schools. At the bottom of the ladder are those people who do not have enough money, do not live in a nice place, and might not have a job. Now think about you—where would you be on the ladder?” Participants were instructed to select which rung best represented their position, with the lowest rung (coded 1) representing “poor” and the highest rung (5) representing “rich”.	Residual score >0
Perceived social support	At age 18, perceived social support was assessed using the Multidimensional Scale of Perceived Social Support (MSPSS), which assesses individuals’ access to supportive relationships with family, friends, and significant others [34]. The 12 items in the MSPSS consist of statements such as “There is a special person who is around when I am in need” and “I can count on my friends when things go wrong”. Participants rated these statements as “not true” (0), “somewhat true” (1), or “very true” (2). We summed the scores to produce an overall social support scale with higher scores reflecting greater social support.	Residual score >0
Education and cognition		
Education	Children’s educational attainment was assessed in the age-18 interview, when children were asked to report their highest educational achievement. Educational attainment was classified following the Qualification and Credit Framework, a credit-based system used in the United Kingdom to assign educational qualifications to a set of ranked levels (http://www.accreditedqualifications.org.uk/qualifications-and-credit-framework-qcf.html). Eighteen-year-olds were classed: as Level 0 if they had no educational qualifications; as Level 1 if they scored a grade of D—G on their General Certificate of Secondary Education (GCSE); as Level 2 if they scored a grade of A*—C on any GCSE; and as Level 3 if they had achieved or were currently working towards university entrance level qualifications (A-levels or equivalent).	Being in category 3 versus 0, 1, 2
Perception of ability to get ahead in life	At age 18, participants were interviewed about their future likelihood of getting ahead in life. A 10-item scale developed for the Office of Juvenile Justice and Delinquency Prevention 3-site study in the United States assessed study members’ expectations for getting ahead by asking about agreement with statements such as “A person like you has a pretty good chance of obtaining a college or university qualification”, “The job market is usually good to people like you”, “Your family can’t give you the opportunities that most people have”, and “As you get older, things will get better” [35]. Statements indicating pessimism about the future (e.g., “You will never have as much opportunity to succeed as other people”) were reverse scored. Scores were summed to create a single score indexing optimism about future labor market prospects, with higher scores indicating greater optimism.	Residual score >0
Cognitive functioning	Executive function and processing speed were assessed with subtests of the Cambridge Neuropsychological Test Automated Battery [36] (including rapid visual information processing (RVP), spatial working memory (SWM) and spatial span subtests) at age 18 years. Scores were scaled to a mean of 100 and SD of 15. Where higher scores indicated poorer cognitive functioning, scores were reverse coded so that for all measures of cognitive function higher scores indicated better functioning (more details in Text S3).	Residual score >0
Physical health		
Physical activity	At age 18, participants completed the Stanford Brief Activity Survey (SBAS). The SBAS contains 2 items, the first item relates to the extent of physical activity engaged in at work, school, or college and the second refers to physical activity during leisure time. Both questions were rated on a 5-point scale: inactive, low intensity, moderate intensity, hard intensity, and very hard intensity. The scales were then combined to derive an overall activity measure [37].	Residual score >0
Sleep quality	Sleep quality was measured using the Pittsburgh Sleep Quality Index (PSQI) [38]. The PSQI consists of 18 self-report items relating to individuals’ sleep patterns and different forms of sleep impairment in the past month. Questions tap a range of aspects of sleep quality and can be used to derive seven component scores (subjective sleep quality, sleep latency, sleep duration, habitual sleep efficiency, sleep disturbances, use of sleep medication, and daytime dysfunction) each scored from 0 to 3. These were summed to produce a global score ranging from 0 to 21 with lower scores reflecting higher sleep quality, and thus scores were reverse coded so that higher scores indicated better sleep quality.	Residual score >0
Pace of biological aging	DunedinPACE was calculated using the R package “DunedinPACE” and is publicly available on GitHub (https://github.com/danbelsky/DunedinPACE) [39]. It was derived from blood samples. DunedinPACE values were standardized to mean = 0, SD = 1. At age 18, estimates of slower biological aging were derived from these values by residualizing for chronological age, and scores were reverse coded so that higher scores indicated slower biological aging.	Residual score >0
Mental well-being		
Life satisfaction	Participants’ life satisfaction was assessed using the Satisfaction with Life Scale [40] with 5 items including “The conditions of my life are excellent” and “I am satisfied with my life”. The response format was a 5-point scale ranging from “strongly disagree” (1) to “strongly agree” (5). The items were summed to create a total score. Higher scores reflected high life satisfaction.	Residual score >0

E-Risk = Environmental Risk Longitudinal Twin Study; SD = standard deviation.

**Table 2:** Characteristics, exposures, and outcomes of children in the whole sample and separately for children who were and were not exposed to any type of victimization

Characteristics	Whole sample (N = 2,066)		Nonvictimized (N = 1,508)		Victimized (N = 558)	
	N	n (%), median [IQR], or mean (SD)	N	n (%), median [IQR], or mean (SD)	N	n (%), median [IQR], or mean (SD)
Biological sex at birth	2,066		1,508		558	
Girls		1,085 (52.5)		799 (53.0)		286 (51.3)
Boys		981 (47.5)		709 (47.0)		272 (48.8)
Family socioeconomic status at age 5	2,066		1,508		558	
Low		691 (33.5)		402 (26.7)		289 (51.8)
Medium		684 (33.1)		531 (35.2)		153 (27.4)
High		691 (33.5)		575 (38.1)		116 (20.8)
Family psychiatric history at age 12	2,010	0.4 (0.3)	1,463	0.3 (0.3)	547	0.5 (0.3)
IQ at age 5	2,052	95.9 (14.6)	1,498	97.5 (14.4)	554	91.4 (14.1)
Victimization (ages 0–12)	2,066		1,508		558	
Exposure to domestic violence	2,066	355 (17.2)	1,508	0	558	355 (63.6)
Bullying by peers	2,062	183 (8.9)	1,504	0	558	183 (32.8)
Physical abuse	2,066	108 (5.2)	1,508	0	558	108 (19.4)
Sexual abuse	2,066	16 (0.8)	1,508	0	558	16 (2.9)
Emotional abuse/neglect	2,066	62 (3.0)	1,508	0	558	62 (11.1)
Physical neglect	2,066	35 (1.7)	1,508	0	558	35 (6.3)
Polyvictimization (ages 0–12 years)	2,066	131 (6.3)	1,508	0	558	131 (23.5)
Victimization for ages 0–12 years retrospectively reported at age 18	2,066	587 (28.4)	1,508	79 (5.2)	558	508 (91.0)
Domains of functioning at age 18						
Social well-being						
Perceived social status	2,042	3.1 (0.7)	1,493	3.2 (0.7)	549	2.9 (0.8)
Perceived social support	2,061	23 [19, 24]	1,505	23 [19, 24]	556	22 [17, 24]
Education and cognition						
Education	2,061		1,506		555	
A levels		1,007 (48.9)		686 (45.6)		368 (66.3)
None or lower qualifications		1,054 (51.1)		820 (54.5)		187 (33.7)
Perception of ability to get ahead in life	2,060	17 [14, 18]	1,503	17 [15, 19]	557	16 [13, 18]
Rapid visual processing A-prime	2,053	100.0 (15.0)	1,499	101.1 (15.0)	554	96.9 (14.7)
Spatial working memory total errors	2,056	66.2 (15.0)	1,500	67.0 (14.9)	556	63.9 (15.1)
Spatial working memory strategy	2,056	41.7 (15.0)	1,500	42.4 (15.2)	556	39.7 (14.4)
Spatial span forward	2,053	100.0 (15.0)	1,499	100.9 (15.1)	554	97.5 (14.3)
Spatial span reversed	2,044	100.0 (15.0)	1,493	101.2 (15.2)	551	96.8 (14.1)
Rapid visual processing mean latency	2,053	104.7 (15.0)	1,499	105.3 (14.9)	554	102.9 (15.0)
Spatial working memory mean time	2,056	301.1 (15.0)	1,500	301.9 (15.3)	556	299.0 (14.1)
Physical health						
Physical activity	2,062	2.8 (1.1)	1,504	2.8 (1.1)	558	2.6 (1.1)
Sleep quality	2,065	13.6 (3.2)	1,507	13.9 (3.0)	558	12.9 (3.4)
Pace of biological aging	1,658	0.3 (0.1)	1,192	0.3 (0.1)	466	0.3 (0.1)
Mental well-being						
Life satisfaction	2,059	20 [17, 22]	1,504	20 [18, 22]	555	19 [16, 21]

Percentages may not total 100 due to rounding.

IQ = intelligence quotient; IQR = interquartile range; SD = standard deviation.

**Table 3:** Associations between exposure to any type of severe victimization and domains of functioning in the whole sample

Domains of functioning^a^	β or odds ratio* (95% CI) unadjusted	*p* value	β or odds ratio* (95% CI) adjusted^b^	*p* value
Social well-being				
Perceived social status	**−0.27** (**−0.35**, **−0.18**)	**<.001**	**−0.11** (**−0.20**, **−0.02**)	**.012**
Perceived social support	**−1.16** (**−1.70**, **−0.63**)	**<.001**	**−0.79** (**−1.35**, **−0.22**)	**.006**
Education and cognition				
Education (ref: none or lower levels)	**0.43** (**0.33**, **0.54**)*	**<.001**	**0.74** (**0.55**, **0.99**)*	**.045**
Perception of ability to get ahead in life	**−1.55** (**−1.95**, **−1.15**)	**<.001**	**−0.90** (**−1.30**, **−0.50**)	**<.001**
Rapid visual processing A-prime	**−4.18** (**−5.79**, **−2.57**)	**<.001**	−1.18 (−2.76, 0.41)	.146
Spatial working memory total errors	**−3.13** (**−4.80**, **−1.46**)	**<.001**	−1.17 (−2.93, 0.58)	.190
Spatial working memory strategy	**−2.69** (**−4.20**, **−1.18**)	**<.001**	−0.96 (−2.57, 0.64)	.239
Spatial span forward	**−3.45** (**−5.01**, **−1.89**)	**<.001**	−1.09 (−2.68, 0.51)	.181
Spatial span reversed	−**4.32** (**−5.89**, **−2.75**)	**<.001**	**−2.26** (**−3.88**, **−0.63**)	**.006**
Rapid visual processing mean latency	−**2.41** (**−3.97**, **−0.84**)	**.003**	−0.44 (−2.14, 1.26)	.611
Spatial working memory mean time	**−2.86** (**−4.46**, **−1.26**)	**<.001**	**−1.68** (**−3.33**, **−0.04**)	**.045**
Physical health				
Physical activity	**−0.17** (**−0.29**, **−0.06**)	**.004**	−0.11 (−0.22, 0.00)	.060
Sleep quality	**−0.94** (**−1.29**, **−0.59**)	**<.001**	**−0.65** (**−1.03**, **−0.27**)	**.001**
Pace of biological aging	**−0.02** (**−0.04**, **−0.01**)	**<.001**	**−0.02** (**−0.03**, **−0.00**)	**.011**
Mental well-being				
Life satisfaction	**−1.63** (**−2.06**, **−1.20**)	**<.001**	**−1.13** (**−1.57**, **−0.69**)	**<.001**

All analyses account for the nonindependence of twin observations.

The n varied from n = 1,605 to n = 2,062, due to different levels of completion of the measures.

Bold text indicates statistically significant result (*p* < .05).

a Each functional outcome was tested in separate linear regression models or with logistic regression for education.

b Adjusted for biological sex at birth, family socioeconomic status at age 5, family history of psychiatric disorders assessed at age 12, and intelligence quotient at age 5.

## Data Availability

The dataset reported in the current article is not publicly available due to lack of informed consent and ethical approval for open access, but is available on request by qualified scientists. Requests require a concept paper describing the purpose of data access, ethical approval at the applicant’s institution, provision for secure data access, and a signed data use agreement (for further details, see here: https://eriskstudy.com/data-access/). Supporting Stata code will become publicly available via F.B.’s GitHub account on publication: https://github.com/FloraBlangis.
