# Supplementary material for: Flourishing in Early Adulthood Among Victimized Children: A Longitudinal Cohort Study
Source: J Adolesc Health. Author manuscript; Available in PMC 2026 Jun 12. (PMC13262664; doi:10.1016/j.jadohealth.2026.02.010)
Supplement: 1 [file NIHMS2178334-supplement-1.docx]

**Supplementary Material**

Blangis F et al. Flourishing in early adulthood among victimised children: A longitudinal cohort study.

**Contents**

| **Supplementary Text 1.** Additional details on the E-Risk Study sample …………………………….. | 2 |
| --- | --- |
| **Supplementary Text 2.** Assessment of victimisation in childhood | 4 |
| **Supplementary Text 3.** Assessment of cognitive functioning at age 18 …………………………….. | 10 |
| **Supplementary Text 4.** Measurement details for confounders ………………………………………. | 12 |
| **Supplementary Figure 1.** Distribution of E-Risk participants at age 18 across Index of Multiple Deprivation deciles ……………………………………………………………………………………. | 13 |
| **Supplementary Figure 2.** Identification of flourishing individuals ………...……………………….. | 14 |
| **Supplementary Figure 3.** Conceptual diagram of the analytic approach ..…………………………... | 15 |
| **Supplementary Table 1.** STROBE checklist ………………………………………………………… | 16 |
| **Supplementary Table 2.** Associations between exposure to any type of victimisation retrospectively reported and domains of functioning in the whole sample .…………………………... | 18 |
| **Supplementary Table 3.** Distribution of flourishing across multiple measures (0–9) (n=558) ……... | 19 |
| **Supplementary Table 4.** Proportion of individuals who are flourishing in early adulthood after exposure to any type of severe victimisation retrospectively reported………..………………………. | 20 |
| **Supplementary Table 5.** Associations between exposure to each type of victimisation and flourishing in the different domains of functioning ………………...…………………………………. | 21 |
| **Supplementary Table 6.** Associations between exposure to poly-victimisation and flourishing in the different domains of functioning ………………………………………………………………….. | 24 |
| **Supplementary Table 7.** Associations between biological sex and flourishing in the different domains of functioning ………………………………………………………………………………... | 25 |
| **Supplementary Table 8.** Associations between exposure to each type of victimisation and flourishing in the different domains of functioning, defining flourishing as a continuous variable…... | 26 |
| **Supplementary Table 9.** Associations between exposure to poly-victimisation and flourishing in the different domains of functioning, defining flourishing as a continuous variable……………………… | 29 |
| **Supplementary Table 10.** Associations between biological sex and flourishing in the different domains of functioning, defining flourishing as a continuous variable……………………………….. | 30 |
| **Supplementary Table 11.** Proportion of individuals who are flourishing in early adulthood after exposure to victimisation in childhood using an enhanced definition of flourishing ………..……….. | 31 |
| **Supplementary Table 12.** Associations between exposure to each type of victimisation and flourishing in the different domains of functioning using an enhanced definition of flourishing, for associations that were statistically significant (at p<0.05) in the previous analyses………………….. | 32 |
| **Supplementary Table 13.** Associations between exposure to poly-victimisation and flourishing in the different domains of functioning using an enhanced definition of flourishing …………………… | 34 |
| **Supplementary references** …………………………………………………………………………... | 35 |

**Supplementary Text 1.** Additional details on the E-Risk Study sample

Participants were members of the Environmental Risk (E-Risk) Longitudinal Twin Study, which tracks the development of a nationally representative birth cohort of 2,232 British twin children. The sample was drawn from a larger birth register of twins born in England and Wales in 1994-1995^1^ Full details about the sample are reported elsewhere.^2^ Briefly, the E-Risk sample was constructed in 1999-2000, when 1,116 families (93% of those eligible) with same-sex 5-year-old twins participated in home-visit assessments. This sample comprised 56% monozygotic (MZ) and 44% dizygotic (DZ) twin pairs; sex was evenly distributed within zygosity (49% male). Families were recruited to represent the UK population of families with newborns in the 1990s, on the basis of residential location throughout England and Wales and mother’s age. Teenaged mothers with twins were over-selected to replace high-risk families who were selectively lost to the register through non-response. Older mothers having twins via assisted reproduction were under-selected to avoid an excess of well-educated older mothers.

Follow-up home-visits were conducted when children were aged 7, 10, 12 and 18 (participation rates were 98%, 96%, 96% and 93%, respectively). Home-visits at ages 5, 7, 10, and 12 years included assessments with participants as well as their mother (or primary caregiver); the home-visit at age 18 included interviews only with the participants. Each participant in a twin pair was assessed by a different interviewer. There were 2,066 E-Risk participants who were assessed at age 18. The average age of the participants at the time of the assessment was 18.4 years (SD = 0.36); all interviews were conducted after the 18th birthday. There were no differences between those who did and did not take part at age 18 in terms of socioeconomic status (SES) assessed when the cohort was initially defined (χ^2^ = 0.86, *p* = 0.65), age-5 intelligence quotient (IQ) scores (t = 0.98, *p* = 0.33), age-5 internalising or externalising behaviour problems (t = 0.40, *p* = 0.69 and t = 0.41, *p* = 0.68, respectively), or childhood poly-victimisation (z=0.51, *p* = 0.61). The study sample represents the full range of socioeconomic conditions in Great Britain, as reflected in the families’ distribution on a neighbourhood-level socioeconomic index (ACORN [A Classification of Residential Neighbourhoods] developed by CACI Inc. for commercial use.^3^ E-Risk families ACORN distribution closely matches that of households nation-wide: 25.6% of E-Risk families live in “wealthy achiever” neighbourhoods compared to 25.3% of households nation-wide; 5.3% vs 11.6% live in “urban prosperity” neighbourhoods; 29.6% vs 26.9% live in “comfortably off” neighbourhoods; 13.4% vs 13.9% live in “moderate means” neighbourhoods; and 26.1% vs 20.7% live in “hard-pressed” neighbourhoods. E-Risk underrepresents urban prosperity neighbourhoods because such households are likely to be childless. Likewise, E-Risk families’ addresses are a near-perfect match to the deciles of the UK’s 2015 Lower-layer Super Output Area (LSOA) Index of Multiple Deprivation (IMD) which averages 1,500 residents; approximately 10% of the E-Risk cohort fills each of the IMD’s 10% bands, indicating that the E-Risk cohort accurately represents the distribution of deprivation in the UK (see **Supplementary Figure 1**).

Parents gave informed consent and twins gave assent between 5-12 years and then informed consent at age 18. The Joint South London and Maudsley and the Institute of Psychiatry Research Ethics Committee approved each phase of the study (NRES 1997/122).

**Supplementary Text 2.** Assessment of victimisation in childhood

We have previously reported evidence on the reliability and validity of our measurement of childhood victimisation.^4^ Here we summarise the method. A team of interviewers visited each family at home when the twins reached ages 5, 7, 10 and 12 years. Each home-visit interview was guided by a series of questions in a booklet. Based on these interviews with the mothers, each interviewer coded in the booklet her initial impression of whether or not she thought a child had been maltreated. The interviewers also recorded notes about their experiences in the home, and if an interviewer was worried about a child, she met with the fieldwork coordinator to debrief. Sometimes, the Study had to make a referral to help a child. Codes, notes, and the fieldwork coordinator’s narratives from the debriefs have been saved over the years to create a dossier for each child with cumulative information about exposure to domestic violence between the mother and her partner; frequent bullying by peers; physical maltreatment by an adult; sexual abuse; emotional abuse and neglect; and physical neglect. All the component measures are outlined briefly below.

Physical domestic violence. Mothers reported about perpetration by and victimisation of 12 forms of physical violence (e.g., slapping, hitting, kicking, strangling) from the Conflict Tactics Scale (CTS),^5^ on three assessment occasions during the child’s first decade of life (when the children were 5, 7, and 10 years of age). Reports of either perpetration or victimisation constituted evidence of physical domestic violence. The CTS has between-partner inter-rater reliabilities of 0.76 for perpetration and 0.82 for victimisation.^6^ Families in which no physical violence took place were coded as 0 (55.2%); families in which physical violence took place on one occasion were coded as 1 (28.0%); and families in which physical violence took place on multiple occasions were coded as 2 (16.8%).

Bullying by peers. Experiences of victimisation by bullies were assessed using both mothers’ and children’s reports. During the interview, the following standard definition of bullying was read out: “Someone is being bullied when another child (a) says mean and hurtful things, makes fun, or calls a person mean and hurtful names; (b) completely ignores or excludes someone from their group of friends or leaves them out on purpose; (c) hits, kicks, or shoves a person, or locks them in a room; (d) tells lies or spreads rumours about them; and (e) other hurtful things like these. We call it bullying when these things happen often, and when it is difficult to make it stop. We do not call it bullying when it is done in a friendly or playful way.” Mothers were interviewed when children were 7, 10, and 12 years old and asked whether either twin had been bullied by another child, responding never, yes, or frequently. We combined mothers’ reports at child age 7 and 10 to derive a measure of victimisation during primary school. Mothers’ reports when the children were 12 years old indexed victimisation during secondary school. During private interviews with the children when they were 12 years old, the children indicated whether they had been bullied by another child during primary or secondary school. When a mother or a child reported victimisation, the interviewer asked them to describe what happened. Notes taken by the interviewers were later checked by an independent rater to verify that the events reported could be classified as instances of bullying operationally defined as evidence of (a) repeated harmful actions, (b) between children, and (c) where there is a power differential between the bully and the victim. Although inter-rater reliability between mothers and children was only modest (kappa = 0.20–0.29), reports of victimisation from both informants were similarly associated with children’s emotional and behavioural problems, suggesting that each informant provides a unique but meaningful perspective on bullying involvement.^7^ We thus combined mother and child reports of victimisation to capture all instances of bullying victimisation for primary and secondary school separately: reported as not victimised by both mother and child; reported by either mother or child as being occasionally victimised; and reported as being occasionally victimised by both informants or as frequently victimised by either mother or child or both.^8^ We then combined these primary and secondary school ratings to create a bullying victimisation variable for the entire childhood period (5–12 years). Children who were never bullied in primary or secondary school or occasionally bullied during one of these time periods were coded as 0 (55.5%); children who were occasionally bullied during primary and secondary school, or frequently bullied during one of these time periods were coded as 1 (35.6%); and children who were frequently bullied at both primary and secondary school were coded as 2 (8.9%).

Physical and sexual harm by an adult. When the twins were aged 5, 7, 10 and 12, their mothers were interviewed about each twins’ experience of intentional harm by an adult. At age 5 we used the standardised clinical protocol from the MultiSite Child Development Project.^9, 10^ At ages 7, 10, and 12 this interview was modified to expand its coverage of contexts for child harm. Interviews were designed to enhance mothers’ comfort with reporting valid child maltreatment information, while also meeting researchers’ responsibilities for referral under the U.K. Children Act. Specifically, mothers were asked whether either of their twins had been intentionally harmed (physically or sexually) by an adult or had contact with welfare agencies. If caregivers endorsed a question, research workers made extensive notes on what had happened, and indicated whether physical and/or psychological harm had occurred. Under the U.K. Children Act, our responsibility was to secure intervention if maltreatment was current and ongoing. Such intervention on behalf of E-Risk families was carried out with parental cooperation in all but one case. No families left the study following intervention. Over the years of data collection, the study developed a cumulative profile for each child, comprising the caregiver reports, recorded debriefings with research workers who had coded any indication of maltreatment at any of the successive home visits, recorded narratives of the successive caregiver interviews, and information from clinicians whenever the Study team made a child-protection referral. The profiles were reviewed at the end of the age–12 phase by two clinical psychologists. Inter-rater agreement between the coders was 90% for cases for whom maltreatment was identified (100% for cases of sexual abuse), and discrepantly coded cases were resolved by consensus review. These were coded as: 0 = no physical harm at any age; 1 = probable physical harm at any age; and 2 = definite physical harm at any age. There were 15.0% of children coded as probably being exposed to physical harm and 5.1% as definitely physically harmed by 12 years of age. There were 1.5% of the children coded as being exposed to sexual abuse.

Emotional abuse and neglect were coded from research workers’ narratives of the home visits at ages 5, 7, 10, and 12. We coded quite severe examples of parental behaviour observed. For example, a mother who had schizophrenia screamed and swore at the children throughout the home visit. As another example, a father who was drunk during the home visit repeatedly spoke abusively to the children in front of the research workers. We found that coders could not empirically separate emotional abuse and emotional neglect in a reliable way and thus such experiences were coded together as emotional abuse/neglect. Inter-rater agreement between the coders exceeded 85% for cases with emotional abuse and neglect, and discrepant cases were resolved by consensus review. Children with no evidence of emotional abuse/neglect were coded as 0 (88.3%), those where there was some indication of emotionally inappropriate/potentially abusive or neglectful behaviour were coded as 1 (8.7%), and where there was evidence of severe emotional abuse/neglect the children were coded as 2 (3.0%).

Physical neglect. The cumulative observations of the physical state of the home environment documented by the research workers during home visits to the twins at ages 5, 7, 10 and 12 were reviewed by two raters for evidence of physical neglect. This was defined as any sign that the caretaker was not providing a safe, sanitary, or healthy environment for the child. This included the child not having proper clothing or food, as well as grossly unsanitary home environments. (However, this did not include a family living in a deprived or crime-ridden neighbourhood). Inter-rater agreement between the coders was 85%, and discrepantly coded cases were resolved by consensus review. Children with no evidence of physical neglect were coded as 0 (90.9%), those for whom there was an indication of minor physical neglect were coded as 1 (7.1%), and where there was evidence of severe physical neglect the children were coded as 2 (2.0%).

Any severe victimisation. Exposure to any severe victimisation was defined as experiencing one or more types of victimisation that received a code of ‘2’ from the following list: physical abuse, sexual abuse, emotional abuse and neglect, physical neglect, exposure to domestic violence, or bullying by peers. There were 1,508 (73.0%) of children who experienced no severe victimisation and 558 (27.0%) who experienced any type of severe victimisation by age 12.

Childhood poly-victimisation. Finkelhor et al. operationalise poly-victimisation as the total number of victimisation types that a child experiences.^11^ The E-Risk poly-victimisation variable was derived by summing all victimisation experiences that received a code of ‘2’. For the current analysis, we dichotomised the poly-victimisation variable into those children who experienced two or more types of severe victimisation before age 12 (N = 131, 6.3%) compared to one or none (N = 1,346, 93.7%).

Retrospective self-reports. Maltreatment was measured retrospectively using the Childhood Trauma Questionnaire (CTQ) when E-Risk participants were aged 18.^12^ The CTQ is a 25-item questionnaire used for retrospective recall of five forms of maltreatment, and has high inter-rater reliability and construct and convergent validity.^13^ The CTQ is also one of the most commonly used retrospective measures of childhood maltreatment, thus increasing the comparability of the present study with previous and future research. Participants reported on their personal experiences of physical, sexual and emotional abuse, and physical and emotional neglect for the period before they were 12 years old (i.e., before entering secondary school). Almost all (99.5%; N = 2,055) E-Risk participants who took part in the age-18 assessment completed the CTQ. Maltreatment scores were dichotomised following CTQ guidelines^12^ to represent none/low (0) versus moderate/severe (1) maltreatment. To allow retrospective self-reports of maltreatment to be compared to prospective informant-reports, emotional abuse and emotional neglect were combined so that a moderate/severe score for emotional abuse and/or emotional neglect represented a moderate/severe score for ‘emotional abuse/neglect’. For comparability to the prospective measure of any severe victimisation, we added domestic violence and bullying by peers from the prospective report to the retrospectively “self-reported maltreatment” variable.

**Supplementary Text 3.** Assessment of cognitive functioning at age 18

Executive function and processing speed were assessed with subtests of the Cambridge Neuropsychological Test Automated Battery^14^ (see below) at age 18 years. Scores were scaled to a mean of 100 and SD of 15.

*Executive functions*

**Rapid Visual Processing A-prime:** Signal-detection measure that taps sustained attention, often called attentional vigilance. The participant scans for a 3-digit target sequence in a digit stream that is ongoing for 7 minutes, and responds whenever a target sequence is spotted. At the most difficult level, the participant scans simultaneously for two target sequences. Higher scores are better.

**Spatial Working Memory Total Errors:** This measure assesses capacity to hold information about spatial location in active memory while searching for information. At the most difficult level, participants memorise 10 locations in one problem. Lower scores are better.

**Spatial Working Memory Strategy:** This measure records trials on which the participant applied a problem-solving strategy by opening boxes in a systematic sequence. Lower scores are better (fewer non-strategic trials).

**Spatial Span Forward:** This measure is the visual non-verbal equivalent of the oral-auditory test Digit Span forward, and measures working memory. At the most difficult level, participants memorise a sequence of 9 coloured stimuli. Higher scores are better.

**Spatial Span Reversed:** This measure is the visual non-verbal equivalent of the oral-auditory Digit Span backward test. Higher scores are better.

*Visual-motel processing speed*

**Rapid Visual Processing Mean Latency** measures the mean latency of response across target signals on the Rapid Visual Processing vigilance task, and reflects reaction time to the visual targets. Lower scores are better (faster).

**Spatial Working Memory Mean Time** measures the mean time to last response across trials, and reflects how rapidly participants solved visual spatial working memory problems. Lower scores are better (faster).

**Supplementary Text 4.** Measurement details for confounders

We adjusted for confounders including biological sex, early childhood IQ, family socioeconomic status (SES), and family psychiatric history, because these factors are known risk factors for childhood victimisation^4,15^ and also influence functional outcomes in early adulthood.^16,17^ The biological sex of the child was reported by mothers at birth. At age 5, children’s IQ was individually tested using a short form of the Wechsler Preschool and Primary Scale of Intelligence–Revised.^18^ Two subtests (Vocabulary and Block Design) were used to prorate children’s IQs following procedures described by Sattler^19^ and then standardised with a mean of 100 and standard deviation of 15. Family SES was measured via a composite of total household income, highest maternal/paternal education, and highest maternal/paternal occupation when children were aged 5. These three indicators were highly correlated (r’s ranged from 0.57-0.67, *p*’s<.05) and loaded significantly onto one latent factor (factor loadings=0.80, 0.70, and 0.83 for income, education, and occupation, respectively). This latent variable was then categorised into tertiles (i.e., low-, medium-, and high-SES).^20^ In private interviews when the children were aged 12, mothers reported on family history of DSM disorders,^21^ which was converted to a proportion (0–1.0) of family members with a history of psychiatric disorders.^22^

**Supplementary Figure 1.** Distribution of E-Risk participants at age 18 across Index of Multiple Deprivation deciles

*Note*. The UK Ministry of Housing, Communities & Local Government Index of Multiple Deprivation is an official measure of relative deprivation for every LSOA small area (approximately 1,500 residents or 650 households each) in England.

**
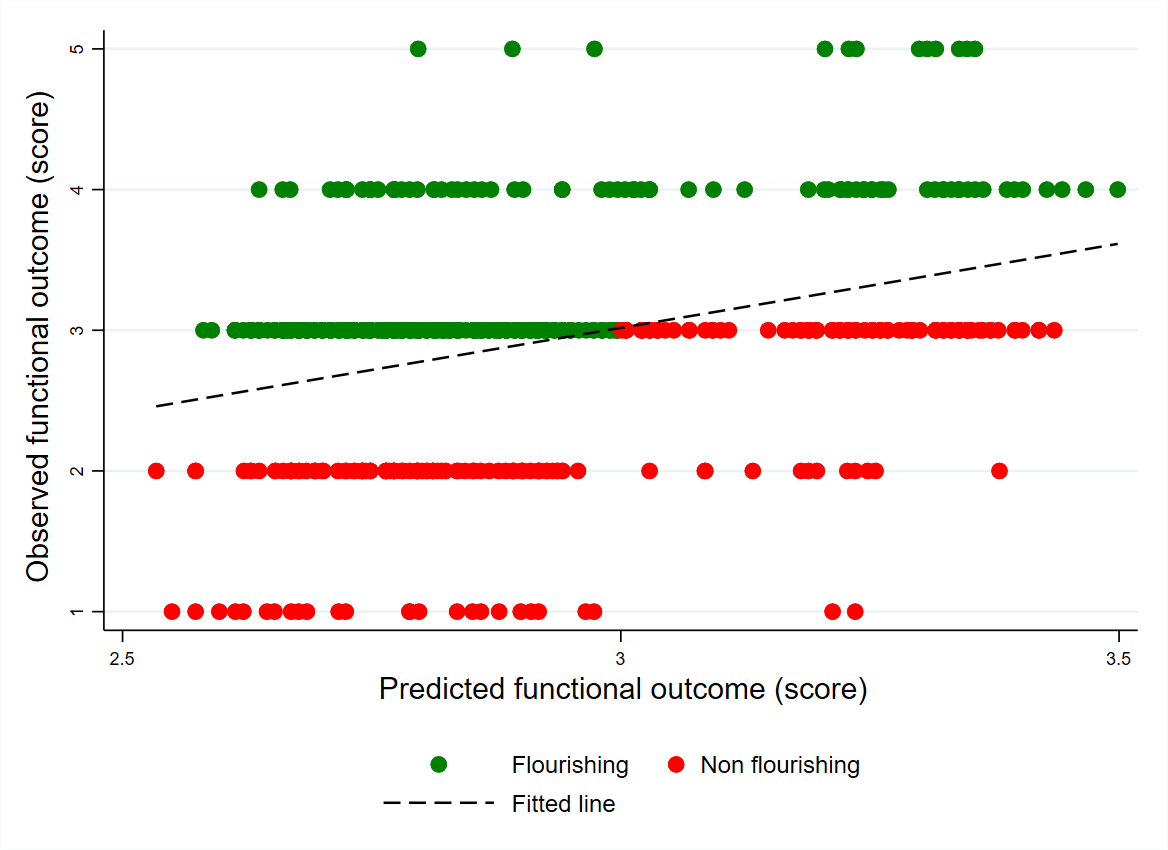
**

**Supplementary Figure 2.** Identification of flourishing individuals

This figure illustrates the method used to identify children exposed to severe victimisation who are functioning better than expected at age 18. The dashed line represents the fitted regression line derived from the model. *Flourishing* individuals (green) are those with positive standardised residuals (>0), meaning their score falls above the fitted regression line. *Non-flourishing* individuals (red) have zero or negative standardised residuals (<0), their score falls on the fitted line or below it.

Any type of severe victimisation

Flourishing

Not flourishing

Residuals > 0*

Residuals ≤ 0

**A**

Each type of victimisation (e.g., physically abused vs. other types)

Flourishing

Not flourishing

Residuals > 0*

Residuals ≤ 0

**B**

Polyvictimised vs. single type of victimisation

Flourishing

Not flourishing

Residuals > 0*

Residuals ≤ 0

**C**

Biological sex
(male vs. female)

Flourishing

Not flourishing

Residuals > 0*

Residuals ≤ 0

**D**

**Supplementary Figure 3.** Conceptual diagram of the analytic approach

* For continuous outcome variables we used the standardised residuals obtained from the multiple regression analyses, which indicate the difference between actual and predicted scores on each outcome variable based on any severe victimisation exposure between 0-12 years of age. Victimised children with residuals >0 (their actual score was greater than their predicted score) were classified as 'flourishing' on that outcome measure, while those scoring 0 (no difference between actual and predicted score) or <0 (actual score was worse than predicted score) were classified as 'not flourishing’ on that outcome measure. For the only categorical outcome, educational attainment, children who achieved one or more A levels were classified as flourishing (best possible outcome at this age), while those with no qualifications or only secondary school qualifications (GCSEs) were classified as not flourishing. All analyses depicted in panels B-D were conducted within the group of participants who were exposed to any severe victimisation in childhood (between 0-12 years of age).

**Supplementary Table 1.** STROBE checklist

|  | Item No | Recommendation | Reported in paragraph # (page) |
| --- | --- | --- | --- |
| **Title and abstract** | 1 | (*a*) Indicate the study’s design with a commonly used term in the title or the abstract | Title and abstract |
|  |  | (*b*) Provide in the abstract an informative and balanced summary of what was done and what was found | Abstract (1) |
| Introduction | | |  |
| Background/rationale | 2 | Explain the scientific background and rationale for the investigation being reported | Introduction (3 and 4)) |
| Objectives | 3 | State specific objectives, including any prespecified hypotheses | Introduction (4 and 5) |
| Methods | | |  |
| Study design | 4 | Present key elements of study design early in the paper | Study Cohort (5) and supplementary text 1 |
| Setting | 5 | Describe the setting, locations, and relevant dates, including periods of recruitment, exposure, follow-up, and data collection | Study Cohort (5) and supplementary text 1 |
| Participants | 6 | (*a*) Give the eligibility criteria, and the sources and methods of selection of participants | Study Cohort (5) and supplementary text 1 |
| Variables | 7 | Clearly define all outcomes, exposures, predictors, potential confounders, and effect modifiers. Give diagnostic criteria, if applicable | Measures (6 and 7), Table 1, and supplementary texts 2, 3, and 4) |
| Data sources/ measurement | 8* | For each variable of interest, give sources of data and details of methods of assessment (measurement). Describe comparability of assessment methods if there is more than one group | Measures (6 and 7), Table 1, and supplementary texts 2, 3, and 4) |
| Bias | 9 | Describe any efforts to address potential sources of bias | - |
| Study size | 10 | Explain how the study size was arrived at | Study Cohort (5 and supplementary text 1) |
| Quantitative variables | 11 | Explain how quantitative variables were handled in the analyses. If applicable, describe which groupings were chosen and why | - |
| Statistical methods | 12 | (*a*) Describe all statistical methods, including those used to control for confounding | Statistical analyses (7 and 8), supplementary Figures 1 and 3 |
|  |  | (*b*) Describe any methods used to examine subgroups and interactions | - |
|  |  | (*c*) Explain how missing data were addressed | - |
|  |  | (*d*) If applicable, describe analytical methods taking account of sampling strategy | - |
|  |  | (*e*) Describe any sensitivity analyses | Statistical analyses (7 and 8) |
| Results | | |  |
| Participants | 13* | (a) Report numbers of individuals at each stage of study—eg numbers potentially eligible, examined for eligibility, confirmed eligible, included in the study, completing follow-up, and analysed | Supplementary text 1 |
|  |  | (b) Give reasons for non-participation at each stage | - |
|  |  | (c) Consider use of a flow diagram | - |
| Descriptive data | 14* | (a) Give characteristics of study participants (eg demographic, clinical, social) and information on exposures and potential confounders | “Is exposure to victimisation during childhood associated with poorer functional outcomes at age 18?” and Table 2 (9) |
|  |  | (b) Indicate number of participants with missing data for each variable of interest | Table 2 |
| Outcome data | 15* | Report numbers of outcome events or summary measures | Tables 2-3 and Figure 1 |
| Main results | 16 | (*a*) Give unadjusted estimates and, if applicable, confounder-adjusted estimates and their precision (eg, 95% confidence interval). Make clear which confounders were adjusted for and why they were included | “Is exposure to victimisation during childhood associated with poorer functional outcomes at age 18?”, “Are some individuals exposed to victimisation in childhood able to flourish when they reach adulthood?” (9) and Figure 1 |
|  |  | (*b*) Report category boundaries when continuous variables were categorized | - |
|  |  | (*c*) If relevant, consider translating estimates of relative risk into absolute risk for a meaningful time period | - |
| Other analyses | 17 | Report other analyses done—eg analyses of subgroups and interactions, and sensitivity analyses | “Does flourishing vary by type of victimisation experienced?”, “Does flourishing vary by exposure to single versus multiple types of victimisation?”, “Does flourishing vary by biological sex?”, and “Sensitivity analyses” (10 and 11) |
| Discussion | | |  |
| Key results | 18 | Summarise key results with reference to study objectives | Discussion section (11 and 12) |
| Limitations | 19 | Discuss limitations of the study, taking into account sources of potential bias or imprecision. Discuss both direction and magnitude of any potential bias | Strengths and limitations (13 and 14) |
| Interpretation | 20 | Give a cautious overall interpretation of results considering objectives, limitations, multiplicity of analyses, results from similar studies, and other relevant evidence | Implications (14) |
| Generalisability | 21 | Discuss the generalisability (external validity) of the study results | Strengths and limitations (13 and 14) |
| Other information | | |  |
| Funding | 22 | Give the source of funding and the role of the funders for the present study and, if applicable, for the original study on which the present article is based | Funding (15) |

**Supplementary Table 2.** Associations between exposure to any type of victimisation retrospectively reported and domains of functioning in the whole sample

| **Domains of functioning^a^** | **Odds ratio* or β (95% CI) unadjusted** | ***P* value** | **Odds ratio* or β (95% CI) adjusted^b^** | ***P* value** |
| --- | --- | --- | --- | --- |
| **Social well-being** |  |  |  |  |
| Perceived social status | **-0.31 (-0.40, -0.23)** | **<0.001** | **-0.18 (-0.26, -0.10)** | **<0.001** |
| Perceived social support | **-1.92 (-2.45, -1.39)** | **<0.001** | **-1.66 (-2.22, -1.10)** | **<0.001** |
| **Education and cognition** |  |  |  |  |
| Education (ref: none or lower levels) | **0.45 (0.35, 0.56)*** | **<0.001** | **0.74 (0.56, 0.98)*** | **0.036** |
| Perception of ability to get ahead in life | **-1.75 (-2.13, -1.36)** | **<0.001** | **-1.18 (-1.55, -0.80)** | **<0.001** |
| Rapid Visual Processing A-prime | **-4.05 (-5.60, -2.51)** | **<0.001** | -1.16 (-2.69, 0.36) | 0.135 |
| Spatial Working Memory Total Errors | **-2.55 (-4.14, -0.96)** | **0.002** | -0.71 (-2.36, 0.94) | 0.400 |
| Spatial Working Memory Strategy | **-2.31 (-3.81, -0.81)** | **0.003** | -0.66 (-2.20, 0.88) | 0.401 |
| Spatial Span Forward | **-3.29 (-4.77, -1.82)** | **<0.001** | -1.11 (-2.63, 0.40) | 0.150 |
| Spatial Span Reversed | **-3.84 (-5.34, -2.34)** | **<0.001** | **-1.72 (-3.26, -0.19)** | **0.028** |
| Rapid Visual Processing Mean Latency | **-2.65 (-4.20, -1.10)** | **0.001** | -0.89 (-2.56, 0.77) | 0.292 |
| Spatial Working Memory Mean Time | **-1.88 (-3.39, -0.37)** | **0.015** | -0.84 (-2.37, 0.69) | 0.283 |
| **Physical health** |  |  |  |  |
| Physical activity | **-0.18 (-0.29, -0.06)** | **0.002** | -0.11 (-0.21, 0.00) | 0.057 |
| Sleep quality | **-1.15 (-1.50, -0.81)** | **<0.001** | **-0.92 (-1.29, -0.55)** | **<0.001** |
| Pace of biological ageing | **-0.02 (-0.04, -0.01)** | **<0.001** | **-0.02 (-0.03, -0.00)** | **0.006** |
| **Mental well-being** |  |  |  |  |
| Life satisfaction | **-1.96 (-2.39, -1.54)** | **<0.001** | **-1.54 (-1.98, -1.11)** | **<0.001** |
| ^a^ Each functional outcome was tested in separate linear regression models or with logistic regression for education.  ^b^ Adjusted for biological sex at birth, family socioeconomic status at age 5, family history of psychiatric disorders assessed at age 12, and intelligence quotient at age 5.  CI, confidence interval.  All analyses account for the non-independence of twin observations.  In the analyses, the n varied from n=1,605 to n=2,065, due to different levels of completion of the measures.  Bold text indicates statistically significant result (p < 0.05). | | | | |

**Supplementary Table 3.** Distribution of flourishing across multiple measures (0–9) (n=558)

| **Number of measures in which victimised individuals flourish** | **n** | **%** |
| --- | --- | --- |
| **0** | 13 | 2.3 |
| **1** | 31 | 5.6 |
| **2** | 41 | 7.4 |
| **3** | 77 | 13.8 |
| **4** | 89 | 16.0 |
| **5** | 119 | 21.3 |
| **6** | 90 | 16.1 |
| **7** | 58 | 10.4 |
| **8** | 34 | 6.1 |
| **9** | 6 | 1.1 |

**Supplementary Table 4.** Proportion of individuals who are flourishing in early adulthood after exposure to any type of severe victimisation retrospectively reported

|  | **Flourishing** | |
| --- | --- | --- |
| **Domains of functioning** | **Yes**  **n (%)** | **No**  **n (%)** |
| **Social well-being** |  |  |
| Perceived social status | 344 (61.8) | 213 (38.2) |
| Perceived social support | 349 (62.0) | 214 (38.0) |
| **Education and cognition** |  |  |
| Education (A levels)^a^ | 203 (34.8) | 381 (65.2) |
| Perception of ability to get ahead in life | 327 (57.9) | 238 (42.1) |
| Spatial Span Reversed | 240 (42.8) | 321 (57.2) |
| Spatial Working Memory Mean Time | 324 (57.4) | 240 (42.6) |
| **Physical health** |  |  |
| Sleep quality | 333 (58.9) | 232 (41.1) |
| Pace of biological ageing | 248 (52.2) | 227 (47.8) |
| **Mental well-being** |  |  |
| Life satisfaction | 320 (56.8) | 243 (43.2) |
| Flourishing was defined using standardised residuals. As a result, analyses included only individuals with complete data for each regression, and sample sizes therefore varied across outcomes. | | |

**Supplementary Table 5.** Associations between exposure to each type of victimisation and flourishing in the different domains of functioning

|  | **Domestic violence** | | | |  | **Bullying by peers** | | | |
| --- | --- | --- | --- | --- | --- | --- | --- | --- | --- |
| **Domains of functioning^a^** | **Flourishing / exposed to domestic violence n (%)** | **Flourishing / exposed to other types of victimisation n (%)** | **OR (95% CI) adjusted^b^ (Ref=other types of victimisation)** | **P value** |  | **Flourishing / exposed to bullying by peers n (%)** | **Flourishing / exposed to other types of victimisation n (%)** | **OR (95% CI) adjusted^b^ (Ref=other types of victimisation)** | **P value** |
| **Social well-being** |  |  |  |  |  |  |  |  |  |
| Perceived social status | 207 (61.1) | 121 (61.7) | 1.03 (0.67, 1.58) | 0.881 |  | 106 (60.6) | 222 (61.7) | 0.86 (0.57, 1.31) | 0.492 |
| Perceived social support | 224 (65.1) | 118 (59.9) | 1.30 (0.86, 1.96) | 0.220 |  | 98 (55.4) | 244 (67.0) | **0.59 (0.39, 0.90)** | **0.015** |
| **Education and cognition** |  |  |  |  |  |  |  |  |  |
| Education | 116 (32.8) | 71 (35.3) | 0.83 (0.52, 1.35) | 0.463 |  | 59 (32.4) | 128 (34.3) | 1.03 (0.64, 1.65) | 0.911 |
| Perception of ability to get ahead in life | 199 (57.9) | 113 (56.8) | 1.05 (0.71, 1.57) | 0.795 |  | 91 (50.8) | 221 (60.7) | **0.66 (0.44, 0.98)** | **0.037** |
| Spatial Span Reversed | 145 (42.3) | 86 (43.9) | 0.89 (0.61, 1.30) | 0.556 |  | 88 (49.7) | 143 (39.5) | **1.61 (1.10, 2.35)** | **0.014** |
| Spatial Working Memory Mean Time | 198 (57.6) | 110 (55.6) | 1.08 (0.72, 1.61) | 0.709 |  | 110 (61.8) | 198 (54.4) | 1.37 (0.93, 2.02) | 0.109 |
| **Physical health** |  |  |  |  |  |  |  |  |  |
| Sleep quality | 209 (60.8) | 109 (54.8) | 1.24 (0.84, 1.83) | 0.274 |  | 96 (53.6) | 222 (61.0) | 0.76 (0.52, 1.11) | 0.157 |
| Pace of biological ageing | 149 (52.1) | 86 (52.2) | 1.02 (0.66, 1.56) | 0.945 |  | 71 (46.7) | 164 (54.3) | 0.75 (0.49, 1.13) | 0.163 |
| **Mental well-being** |  |  |  |  |  |  |  |  |  |
| Life satisfaction | 190 (55.2) | 116 (58.9) | 0.90 (0.60, 1.33) | 0.593 |  | 102 (57.6) | 204 (56.0) | 1.03 (0.68, 1.54) | 0.900 |
| ^a^ Each functional outcome was tested in separate binary logistic regression models; ^b^ Adjusted for biological sex at birth, family socioeconomic status at age 5, family history of psychiatric disorders assessed at age 12, and intelligence quotient at age 5.  CI, confidence interval; OR, odds ratio.  All analyses account for the non-independence of twin observations.  Flourishing was defined using standardised residuals. As a result, analyses included only individuals with complete data for each regression, and sample sizes therefore varied across outcomes.  Bold text indicates statistically significant result (p < 0.05). | | | | | | | | | |

**Supplementary Table 5 (continued)**

|  | **Physical abuse** | | | |  | **Sexual abuse** | | | |
| --- | --- | --- | --- | --- | --- | --- | --- | --- | --- |
| **Domains of functioning^a^** | **Flourishing / exposed to physical abuse n (%)** | **Flourishing / exposed to other types of victimisation n (%)** | **OR (95% CI) adjusted^b^ (Ref=other types of victimisation)** | **P value** |  | **Flourishing / exposed to sexual abuse n (%)** | **Flourishing / exposed to other types of victimisation n (%)** | **OR (95% CI) adjusted^b^ (Ref=other types of victimisation)** | **P value** |
| **Social well-being** |  |  |  |  |  |  |  |  |  |
| Perceived social status | 52 (49.5) | 276 (64.2) | **0.45 (0.26, 0.78)** | **0.004** |  | 11 (73.3) | 317 (61.0) | 1.25 (0.45, 3.43) | 0.671 |
| Perceived social support | 55 (51.9) | 287 (66.0) | **0.61 (0.38, 0.98)** | **0.043** |  | 11 (73.3) | 331 (62.9) | 1.59 (0.49, 5.12) | 0.441 |
| **Education and cognition** |  |  |  |  |  |  |  |  |  |
| Education | 27 (25.3) | 160 (35.7) | 0.78 (0.41, 1.46) | 0.433 |  | 5 (31.3) | 182 (33.8) | 1.62 (0.42, 6.28) | 0.487 |
| Perception of ability to get ahead in life | 57 (53.3) | 255 (58.5) | 0.85 (0.53, 1.37) | 0.497 |  | 8 (53.3) | 304 (57.6) | 0.93 (0.28, 3.15) | 0.913 |
| Spatial Span Reversed | 39 (37.1) | 192 (44.2) | 0.69 (0.43, 1.11) | 0.130 |  | 3 (21.4) | 228 (43.4) | **0.32 (0.11, 0.94)** | **0.039** |
| Spatial Working Memory Mean Time | 56 (52.8) | 252 (57.8) | 0.78 (0.48, 1.27) | 0.320 |  | 10 (66.7) | 298 (56.6) | 1.75 (0.49, 6.21) | 0.384 |
| **Physical health** |  |  |  |  |  |  |  |  |  |
| Sleep quality | 60 (56.1) | 258 (59.2) | 0.84 (0.52, 1.35) | 0.472 |  | 6 (40.0) | 312 (59.1) | 0.45 (0.16, 1.26) | 0.128 |
| Pace of biological ageing | 41 (48.2) | 194 (52.6) | 0.81 (0.47, 1.38) | 0.429 |  | 4 (30.8) | 231 (52.4) | 0.42 (0.12, 1.44) | 0.168 |
| **Mental well-being** |  |  |  |  |  |  |  |  |  |
| Life satisfaction | 50 (47.2) | 256 (58.9) | 0.66 (0.41, 1.08) | 0.100 |  | 4 (26.7) | 302 (57.4) | **0.26 (0.08, 0.82)** | **0.021** |
| ^a^ Each functional outcome was tested in separate binary logistic regression models; ^b^ Adjusted for biological sex at birth, family socioeconomic status at age 5, family history of psychiatric disorders assessed at age 12, and intelligence quotient at age 5.  CI, confidence interval; OR, odds ratio.  All analyses account for the non-independence of twin observations.  Flourishing was defined using standardised residuals. As a result, analyses included only individuals with complete data for each regression, and sample sizes therefore varied across outcomes.  Bold text indicates statistically significant result (p < 0.05). | | | | | | | | | |

**Supplementary Table 5 (continued)**

|  | **Emotional abuse and neglect** | | | |  | **Physical neglect** | | | |
| --- | --- | --- | --- | --- | --- | --- | --- | --- | --- |
| **Domains of functioning^a^** | **Flourishing / exposed to emotional abuse and neglect n (%)** | **Flourishing / exposed to other types of victimisation n (%)** | **OR (95% CI) adjusted^b^ (Ref=other types of victimisation)** | **P value** |  | **Flourishing / exposed to physical neglect n (%)** | **Flourishing / exposed to other types of victimisation n (%)** | **OR (95% CI) adjusted^b^ (Ref=other types of victimisation)** | **P value** |
| **Social well-being** |  |  |  |  |  |  |  |  |  |
| Perceived social status | 27 (45.8) | 301 (63.2) | **0.34 (0.17, 0.70)** | **0.003** |  | 19 (55.9) | 309 (61.7) | 0.56 (0.22, 1.43) | 0.227 |
| Perceived social support | 31 (50.8) | 311 (64.8) | 0.63 (0.33, 1.21) | 0.168 |  | 21 (60.0) | 321 (63.4) | 1.06 (0.45, 2.53) | 0.891 |
| **Education and cognition** |  |  |  |  |  |  |  |  |  |
| Education | 10 (16.1) | 177 (35.9) | 0.57 (0.23, 1.41) | 0.225 |  | 7 (20.0) | 180 (34.6) | 0.89 (0.33, 2.39) | 0.814 |
| Perception of ability to get ahead in life | 32 (52.5) | 280 (58.1) | 0.87 (0.47, 1.59) | 0.647 |  | 16 (45.7) | 296 (58.3) | 0.68 (0.34, 1.38) | 0.287 |
| Spatial Span Reversed | 24 (39.3) | 207 (43.3) | 0.79 (0.43, 1.46) | 0.457 |  | 15 (42.9) | 216 (42.9) | 0.90 (0.42, 1.97) | 0.800 |
| Spatial Working Memory Mean Time | 33 (54.1) | 275 (57.2) | 0.87 (0.48, 1.56) | 0.632 |  | 22 (62.9) | 286 (56.4) | 1.31 (0.55, 3.16) | 0.544 |
| **Physical health** |  |  |  |  |  |  |  |  |  |
| Sleep quality | 32 (52.5) | 286 (59.3) | 0.72 (0.41, 1.27) | 0.260 |  | 19 (54.3) | 299 (58.9) | 0.79 (0.40, 1.56) | 0.495 |
| Pace of biological ageing | 27 (51.9) | 208 (51.7) | 1.00 (0.50, 1.99) | 0.992 |  | 13 (44.8) | 222 (52.2) | 0.74 (0.33, 1.64) | 0.451 |
| **Mental well-being** |  |  |  |  |  |  |  |  |  |
| Life satisfaction | 27 (44.3) | 279 (58.1) | 0.61 (0.31, 1.20) | 0.150 |  | 16 (45.7) | 290 (57.3) | 0.74 (0.34, 1.60) | 0.442 |
| ^a^ Each functional outcome was tested in separate binary logistic regression models; ^b^ Adjusted for biological sex at birth, family socioeconomic status at age 5, family history of psychiatric disorders assessed at age 12, and intelligence quotient at age 5.  CI, confidence interval; OR, odds ratio.  All analyses account for the non-independence of twin observations.  Flourishing was defined using standardised residuals. As a result, analyses included only individuals with complete data for each regression, and sample sizes therefore varied across outcomes.  Bold text indicates statistically significant result (p < 0.05). | | | | | | | | | |

**Supplementary Table 6.** Associations between exposure to poly-victimisation and flourishing in the different domains of functioning

| **Domains of functioning^a^** | **Flourishing / exposed to single victimisation n (%)** | **Flourishing / exposed to poly-victimisation n (%)** | **OR (95% CI) adjusted^b^**  **(Ref=single victimisation)** | **P value** | |
| --- | --- | --- | --- | --- | --- |
| **Social well-being** |  |  |  |  | |
| Perceived social status | 262 (64.1) | 66 (52.4) | **0.45 (0.28, 0.73)** | **0.001** | |
| Perceived social support | 274 (66.3) | 68 (53.1) | 0.64 (0.40, 1.02) | 0.061 | |
| **Education and cognition** |  |  |  |  | |
| Education | 162 (38.2) | 25 (19.1) | 0.56 (0.31, 1.02) | 0.058 | |
| Perception of ability to get ahead in life | 250 (60.4) | 62 (48.1) | **0.64 (0.41, 0.99)** | **0.044** | |
| Spatial Span Reversed | 174 (42.2) | 57 (44.9) | 1.05 (0.67, 1.66) | 0.821 | |
| Spatial Working Memory Mean Time | 234 (56.5) | 74 (57.8) | 1.04 (0.65, 1.66) | 0.865 | |
| **Physical health** |  |  |  |  | |
| Sleep quality | 250 (60.4) | 68 (52.7) | 0.69 (0.45, 1.06) | 0.087 | |
| Pace of biological ageing | 189 (54.8) | 46 (42.2) | **0.57 (0.35, 0.93)** | **0.024** | |
| **Mental well-being** |  |  |  |  | |
| Life satisfaction | 247 (59.8) | 59 (46.1) | **0.61 (0.38, 0.98)** | **0.043** | |
| ^a^ Each functional outcome was tested in separate binary logistic regression models; ^b^ Adjusted for biological sex at birth, family socioeconomic status at age 5, family history of psychiatric disorders assessed at age 12, and intelligence quotient at age 5.  CI, confidence interval; OR, odds ratio.  All analyses account for the non-independence of twin observations.  Flourishing was defined using standardised residuals. As a result, analyses included only individuals with complete data for each regression, and sample sizes therefore varied across outcomes.  Bold text indicates statistically significant result (p < 0.05). | | | | |  |

**Supplementary Table 7.** Associations between biological sex and flourishing in the different domains of functioning

| **Domains of functioning^a^** | **Flourishing in boys n (%)** | **Flourishing in girls n (%)** | **OR (95% CI) adjusted^b^ (Ref=boys)** | **P value** |
| --- | --- | --- | --- | --- |
| **Social well-being** |  |  |  |  |
| Perceived social status | 160 (63.0) | 168 (59.8) | 0.94 (0.63, 1.42) | 0.782 |
| Perceived social support | 156 (60.5) | 186 (65.7) | 1.27 (0.85, 1.90) | 0.251 |
| **Education and cognition** |  |  |  |  |
| Education | 73 (27.0) | 114 (40.0) | **1.61 (1.02, 2.55)** | **0.042** |
| Perception of ability to get ahead in life | 157 (60.4) | 155 (54.8) | 0.77 (0.53, 1.14) | 0.193 |
| Spatial Span Reversed | 107 (41.5) | 124 (44.1) | 1.11 (0.77, 1.61) | 0.569 |
| Spatial Working Memory Mean Time | 148 (57.1) | 160 (56.5) | 0.96 (0.65, 1.41) | 0.839 |
| **Physical health** |  |  |  |  |
| Sleep quality | 154 (59.2) | 164 (58.0) | 0.94 (0.65, 1.36) | 0.736 |
| Pace of biological ageing | 118 (52.0) | 117 (51.5) | 0.97 (0.64, 1.48) | 0.899 |
| **Mental well-being** |  |  |  |  |
| Life satisfaction | 151 (58.5) | 155 (54.8) | 0.87 (0.59, 1.28) | 0.465 |
| ^a^ Each functional outcome was tested in separate binary logistic regression models; ^b^ Adjusted for family socioeconomic status at age 5, family history of psychiatric disorders assessed at age 12, and intelligence quotient at age 5.  CI, confidence interval; OR, odds ratio.  All analyses account for the non-independence of twin observations.  Flourishing was defined using standardised residuals. As a result, analyses included only individuals with complete data for each regression, and sample sizes therefore varied across outcomes.  Bold text indicates statistically significant result (p < 0.05). | | | | |

**Supplementary Table 8.** Associations between exposure to each type of victimisation and flourishing in the different domains of functioning, defining flourishing as a continuous variable

|  | **Domestic violence** | | | | | |  | **Bullying by peers** | | | | | |
| --- | --- | --- | --- | --- | --- | --- | --- | --- | --- | --- | --- | --- | --- |
| **Domains of functioning^a^** | **Flourishing / exposed to domestic violence** | | **Flourishing / exposed to other types of victimisation** | | **β (95% CI) adjusted^b^** | **P value** |  | **Flourishing / exposed to bullying by peers** | | **Flourishing / exposed to other types of victimisation** | | **β (95% CI) adjusted^b^** | **P value** |
|  | **n** | **Mean (SD)** | **n** | **Mean (SD)** |  |  |  | **n** | **Mean (SD)** | **n** | **Mean (SD)** |  |  |
| **Social well-being** |  |  |  |  |  |  |  |  |  |  |  |  |  |
| Perceived social status | 207 | 0.69 (0.65) | 121 | 0.58 (0.62) | 0.08 (-0.06, 0.22) | 0.237 |  | 106 | 0.56 (0.57) | 222 | 0.69 (0.67) | -0.10 (-0.24, 0.29) | 0.126 |
| Perceived social support | 224 | 0.74 (0.31) | 118 | 0.68 (0.31) | 0.06 (-0.01, 0.13) | 0.083 |  | 98 | 0.68 (0.32) | 244 | 0.74 (0.31) | **-0.08 (-0.15, -0.00)** | **0.042** |
| **Education and cognition** |  |  |  |  |  |  |  |  |  |  |  |  |  |
| Perception of ability to get ahead in life | 199 | 0.82 (0.50) | 113 | 0.81 (0.52) | 0.04 (-0.08, 0.16) | 0.544 |  | 91 | 0.79 (0.55) | 221 | 0.82 (0.49) | -0.04 (-0.17, 0.08) | 0.510 |
| Spatial Span Reversed | 145 | 0.84 (0.68) | 86 | 0.94 (0.77) | -0.11 (-0.31, 0.10) | 0.322 |  | 88 | 0.83 (0.70) | 143 | 0.90 (0.73) | -0.07 (-0.26, 0.13) | 0.517 |
| Spatial Working Memory Mean Time | 198 | 0.70 (0.44) | 110 | 0.74 (0.42) | -0.04 (-0.15, 0.07) | 0.487 |  | 110 | 0.73 (0.41) | 198 | 0.71 (0.45) | 0.01 (-0.09, 0.12) | 0.797 |
| **Physical health** |  |  |  |  |  |  |  |  |  |  |  |  |  |
| Sleep quality | 209 | 0.77 (0.45) | 109 | 0.68 (0.43) | 0.10 (-0.02, 0.21) | 0.095 |  | 96 | 0.73 (0.47) | 222 | 0.74 (0.44) | -0.02 (-0.14, 0.10) | 0.718 |
| Pace of biological ageing | 149 | 0.79 (0.66) | 86 | 0.71 (0.56) | 0.10 (-0.11, 0.30) | 0.339 |  | 71 | 0.68 (0.52) | 164 | 0.80 (0.66) | -0.12 (-0.31, 0.07) | 0.205 |
| **Mental well-being** |  |  |  |  |  |  |  |  |  |  |  |  |  |
| Life satisfaction | 190 | 0.90 (0.57) | 116 | 0.64 (0.58) | **0.27 (0.12, 0.41)** | **<0.001** |  | 102 | 0.77 (0.56) | 302 | 0.82 (0.60) | -0.04 (-0.19, 0.11) | 0.574 |
| ^a^ Each functional outcome was tested in separate linear regression models; ^b^ Adjusted for biological sex at birth, family socioeconomic status at age 5, family history of psychiatric disorders assessed at age 12, and intelligence quotient at age 5.  CI, confidence interval.  All analyses account for the non-independence of twin observations.  Flourishing was defined using standardised residuals. As a result, analyses included only individuals with complete data for each regression, and sample sizes therefore varied across outcomes.  Bold text indicates statistically significant result (p < 0.05). | | | | | | | | | | | | | |

**Supplementary Table 8 (continued)**

|  | **Physical abuse** | | | | | | | | |  | | **Sexual abuse** | | | | | | |
| --- | --- | --- | --- | --- | --- | --- | --- | --- | --- | --- | --- | --- | --- | --- | --- | --- | --- | --- |
| **Domains of functioning^a^** | **Flourishing / exposed to physical abuse** | | **Flourishing / exposed to other types of victimisation** | | **β (95% CI) adjusted^b^** | | **P value** | |  | | **Flourishing / exposed to sexual abuse** | | | **Flourishing / exposed to other types of victimisation** | | | **β (95% CI) adjusted^b^** | **P value** |
|  | **n** | **Mean (SD)** | **n** | **Mean (SD)** | |  | |  | |  | | **n** | **Mean (SD)** | | **n** | **Mean (SD)** |  |  |
| **Social well-being** |  |  |  |  | |  | |  | |  | |  |  | |  |  |  |  |
| Perceived social status | 52 | 0.70 (0.73) | 276 | 0.64 (0.62) | | 0.10 (-0.10, 0.31) | | 0.319 | |  | | 11 | 0.38 (0.49) | | 317 | 0.66 (0.64) | -0.18 (-0.49, 0.12) | 0.233 |
| Perceived social support | 55 | 0.75 (0.32) | 287 | 0.72 (0.31) | | -0.00 (-0.10, 0.10) | | 0.937 | |  | | 11 | 0.76 (0.26) | | 331 | 0.72 (0.31) | 0.06 (-0.07, 0.18) | 0.369 |
| **Education and cognition** |  |  |  |  | |  | |  | |  | |  |  | |  |  |  |  |
| Perception of ability to get ahead in life | 57 | 0.83 (0.53) | 255 | 0.81 (0.50) | | -0.03 (-1.18, 0.11) | | 0.652 | |  | | 8 | 0.81 (0.55) | | 304 | 0.82 (0.51) | -0.06 (-0.42, 0.31) | 0.758 |
| Spatial Span Reversed | 39 | 1.01 (0.79) | 192 | 0.85 (0.70) | | 0.18 (-0.12, 0.48) | | 0.246 | |  | | 3 | 0.73 (0.78) | | 228 | 0.88 (0.72) | -0.14 (-0.89, 0.62) | 0.720 |
| Spatial Working Memory Mean Time | 56 | 0.73 (0.43) | 252 | 0.71 (0.44) | | 0.00 (-0.13, 0.14) | | 0.965 | |  | | 10 | 0.80 (0.40) | | 298 | 0.71 (0.44) | 0.07 (-0.24, 0.39) | 0.638 |
| **Physical health** |  |  |  |  | |  | |  | |  | |  |  | |  |  |  |  |
| Sleep quality | 60 | 0.59 (0.42) | 258 | 0.77 (0.44) | | **-0.20 (-0.34, -0.05)** | | **0.008** | |  | | 6 | 0.47 (0.38) | | 312 | 0.74 (0.45) | **-0.31 (-0.61, -0.01)** | **0.040** |
| Pace of biological ageing | 41 | 0.66 (0.72) | 194 | 0.79 (0.60) | | -0.62 (-0.34, 0.17) | | 0.533 | |  | | 4 | 0.96 (1.00) | | 231 | 0.76 (0.62) | 0.20 (-0.70, 1.09) | 0.664 |
| **Mental well-being** |  |  |  |  | |  | |  | |  | |  |  | |  |  |  |  |
| Life satisfaction | 50 | 0.80 (0.59) | 256 | 0.83 (0.57) | | **-0.24 (-0.44, -0.04)** | | **0.019** | |  | | 4 | 0.55 (0.69) | | 302 | 0.81 (0.59) | -0.21 (-0.89, 0.47) | 0.547 |
| ^a^ Each functional outcome was tested in separate linear regression models; ^b^ Adjusted for biological sex at birth, family socioeconomic status at age 5, family history of psychiatric disorders assessed at age 12, and intelligence quotient at age 5.  CI, confidence interval.  All analyses account for the non-independence of twin observations.  Flourishing was defined using standardised residuals. As a result, analyses included only individuals with complete data for each regression, and sample sizes therefore varied across outcomes.  Bold text indicates statistically significant result (p < 0.05). | | | | | | | | | | | | | | | | | | |

**Supplementary Table 8 (continued)**

|  | **Emotional abuse and neglect** | | | | | | | | |  | | **Physical neglect** | | | | | | |
| --- | --- | --- | --- | --- | --- | --- | --- | --- | --- | --- | --- | --- | --- | --- | --- | --- | --- | --- |
| **Domains of functioning^a^** | **Flourishing / exposed to emotional abuse and neglect** | | **Flourishing / exposed to other types of victimisation** | | **β (95% CI) adjusted^b^** | | **P value** | |  | | **Flourishing / exposed to physical neglect** | | | **Flourishing / exposed to other types of victimisation** | | | **β (95% CI) adjusted^b^** | **P value** |
|  | **n** | **Mean (SD)** | **n** | **Mean (SD)** | |  | |  | |  | | **n** | **Mean (SD)** | | **n** | **Mean (SD)** |  |  |
| **Social well-being** |  |  |  |  | |  | |  | |  | |  |  | |  |  |  |  |
| Perceived social status | 27 | 0.45 (0.42) | 301 | 0.66 (0.66) | | -0.12 (-0.30, 0.06) | | 0.207 | |  | | 19 | 0.45 (0.37) | | 309 | 0.66 (0.65) | -0.12 (-0.38, 0.14) | 0.357 |
| Perceived social support | 31 | 0.75 (0.39) | 311 | 0.72 (0.30) | | -0.01 (-0.15, 0.14) | | 0.925 | |  | | 21 | 0.78 (0.30) | | 321 | 0.72 (0.31) | -0.01 (-0.13, 0.12) | 0.921 |
| **Education and cognition** |  |  |  |  | |  | |  | |  | |  |  | |  |  |  |  |
| Perception of ability to get ahead in life | 32 | 0.93 (0.53) | 280 | 0.80 (0.50) | | 0.07 (-0.13, 0.27) | | 0.494 | |  | | 16 | 0.82 (0.49) | | 296 | 0.82 (0.51) | -0.08 (-0.34, 0.19) | 0.558 |
| Spatial Span Reversed | 24 | 0.96 (0.79) | 207 | 0.87 (0.71) | | 0.11 (-0.29, 0.51) | | 0.585 | |  | | 15 | 0.99 (0.90) | | 216 | 0.87 (0.70) | 0.12 (-0.38, 0.63) | 0.630 |
| Spatial Working Memory Mean Time | 33 | 0.75 (0.45) | 275 | 0.71 (0.43) | | -0.01 (-0.20, 0.18) | | 0.938 | |  | | 22 | 0.69 (0.31) | | 286 | 0.72 (0.44) | -0.05 (-0.22, 0.13) | 0.586 |
| **Physical health** |  |  |  |  | |  | |  | |  | |  |  | |  |  |  |  |
| Sleep quality | 32 | 0.65 (0.40) | 286 | 0.75 (0.45) | | -0.11 (-0.27, 0.04) | | 0.150 | |  | | 19 | 0.66 (0.45) | | 299 | 0.74 (0.45) | -0.10 (-0.33, 0.13) | 0.412 |
| Pace of biological ageing | 27 | 0.64 (0.54) | 208 | 0.78 (0.63) | | -0.07 (-0.34, 0.20) | | 0.591 | |  | | 13 | 0.32 (0.26) | | 222 | 0.79 (0.63) | **-0.43 (-0.63, -0.24)** | **<0.001** |
| **Mental well-being** |  |  |  |  | |  | |  | |  | |  |  | |  |  |  |  |
| Life satisfaction | 27 | 0.63 (0.66) | 279 | 0.82 (0.58) | | -0.22 (-0.55, 0.10) | | 0.177 | |  | | 16 | 0.68 (0.75) | | 290 | 0.81 (0.58) | -0.14 (-0.54, 0.26) | 0.487 |
| ^a^ Each functional outcome was tested in separate linear regression models; ^b^ Adjusted for biological sex at birth, family socioeconomic status at age 5, family history of psychiatric disorders assessed at age 12, and intelligence quotient at age 5.  CI, confidence interval.  All analyses account for the non-independence of twin observations.  Flourishing was defined using standardised residuals. As a result, analyses included only individuals with complete data for each regression, and sample sizes therefore varied across outcomes.  Bold text indicates statistically significant result (p < 0.05). | | | | | | | | | | | | | | | | | | |

**Supplementary Table 9.** Associations between exposure to poly-victimisation and flourishing in the different domains of functioning, defining flourishing as a continuous variable

| **Domains of functioning^a^** | **Flourishing / exposed to single victimisation** | | **Flourishing / exposed to poly-victimisation** | | **β (95% CI) adjusted^b^** | **P value** |
| --- | --- | --- | --- | --- | --- | --- |
|  | **n** | **Mean (SD)** | **n** | **Mean (SD)** |  |  |
| **Social well-being** |  |  |  |  |  |  |
| Perceived social status | 262 | 0.67 (0.66) | 66 | 0.55 (0.54) | -0.04 (-0.20, 0.12) | 0.626 |
| Perceived social support | 274 | 0.72 (0.31) | 68 | 0.74 (0.35) | -0.03 (-0.12, 0.06) | 0.586 |
| **Education and cognition** |  |  |  |  |  |  |
| Perception of ability to get ahead in life | 250 | 0.81 (0.50) | 62 | 0.83 (0.55) | -0.04 (-0.19, 0.11 | 0.593 |
| Spatial Span Reversed | 174 | 0.90 (0.72) | 57 | 0.81 (0.71) | -0.09 (-0.32, 0.15) | 0.467 |
| Spatial Working Memory Mean Time | 234 | 0.72 (0.45) | 74 | 0.71 (0.40) | -0.03 (-0.15, 0.09) | 0.659 |
| **Physical health** |  |  |  |  |  |  |
| Sleep quality | 250 | 0.76 (0.45) | 68 | 0.68 (0.43) | -0.09 (-0.22, 0.05) | 0.206 |
| Pace of biological ageing | 189 | 0.79 (0.61) | 46 | 0.66 (0.70) | -0.07 (-0.32, 0.17) | 0.554 |
| **Mental well-being** |  |  |  |  |  |  |
| Life satisfaction | 247 | 0.81 (0.59) | 59 | 0.75 (0.58) | -0.11 (-0.30, 0.09) | 0.286 |
| ^a^ Each functional outcome was tested in separate linear regression models; ^b^ Adjusted for biological sex, family socioeconomic status at age 5, family history of psychiatric disorders assessed at age 12, and intelligence quotient at age 5.  CI, confidence interval.  All analyses account for the non-independence of twin observations.  Flourishing was defined using standardised residuals. As a result, analyses included only individuals with complete data for each regression, and sample sizes therefore varied across outcomes. | | | | | | |

**Supplementary Table 10.** Associations between biological sex and flourishing in the different domains of functioning, defining flourishing as a continuous variable

| **Domains of functioning^a^** | **Flourishing in boys** | | **Flourishing in girls** | | **β (95% CI) adjusted^b^** | **P value** |
| --- | --- | --- | --- | --- | --- | --- |
|  | **n** | **Mean (SD)** | **n** | **Mean (SD)** |  |  |
| **Social well-being** |  |  |  |  |  |  |
| Perceived social status | 160 | 0.63 (0.63) | 168 | 0.66 (0.65) | 0.01 (-0.13, 0.15) | 0.874 |
| Perceived social support | 156 | 0.79 (0.34) | 186 | 0.67 (0.27) | **-0.11 (-0.18, -0.04)** | **0.002** |
| **Education and cognition** |  |  |  |  |  |  |
| Perception of ability to get ahead in life | 157 | 0.81 (0.51) | 155 | 0.82 (0.51) | 0.02 (-0.18, 0.22) | 0.854 |
| Spatial Span Reversed | 107 | 0.85 (0.68) | 124 | 0.90 (0.75) | -0.03 (-0.38, 0.33) | 0.886 |
| Spatial Working Memory Mean Time | 148 | 0.76 (0.46) | 160 | 0.68 (0.41) | -0.08 (-0.19, 0.03) | 0.139 |
| **Physical health** |  |  |  |  |  |  |
| Sleep quality | 154 | 0.74 (0.42) | 164 | 0.74 (0.47) | 0.01 (-0.10, 0.12) | 0.858 |
| Pace of biological ageing | 118 | 0.70 (0.53) | 117 | 0.83 (0.70) | 0.13 (-0.07, 0.33) | 0.213 |
| **Mental well-being** |  |  |  |  |  |  |
| Life satisfaction | 151 | 0.84 (0.61) | 155 | 0.77 (0.57) | -0.07 (-0.21, 0.07) | 0.305 |
| ^a^ Each functional outcome was tested in separate linear regression models; ^b^ Adjusted for family socioeconomic status at age 5, family history of psychiatric disorders assessed at age 12, and intelligence quotient at age 5.  CI, confidence interval; OR, odds ratio.  All analyses account for the non-independence of twin observations.  Flourishing was defined using standardised residuals. As a result, analyses included only individuals with complete data for each regression, and sample sizes therefore varied across outcomes.  Bold text indicates statistically significant result (p < 0.05). | | | | | | |

**Supplementary Table 11.** Proportion of individuals who are flourishing in early adulthood after exposure to victimisation in childhood using an enhanced definition of flourishing

|  | **Flourishing** | |
| --- | --- | --- |
| **Domains of functioning** | **Yes**  **n (%)** | **No**  **n (%)** |
| **Social well-being** |  |  |
| Perceived social status | 93 (17.4) | 442 (82.6) |
| Perceived social support | 217 (40.1) | 324 (59.9) |
| **Education and cognition** |  |  |
| Perception of ability to get ahead in life | 165 (30.4) | 378 (69.6) |
| Spatial Span Reversed | 118 (21.9) | 421 (78.1) |
| Spatial Working Memory Mean Time | 157 (29.0) | 385 (71.0) |
| **Physical health** |  |  |
| Sleep quality | 158 (29.1) | 385 (70.9) |
| Pace of biological ageing | 111 (24.5) | 343 (75.6) |
| **Mental well-being** |  |  |
| Life satisfaction | 148 (27.4) | 393 (72.6) |
| Victimised individuals with standardised residuals in the top 25th percentile were considered to have enhanced flourishing.  Flourishing was defined using standardised residuals. As a result, analyses included only individuals with complete data for each regression, and sample sizes therefore varied across outcomes. | | |

**Supplementary Table 12.** Associations between exposure to each type of victimisation and flourishing in the different domains of functioning using an enhanced definition of flourishing, for associations that were statistically significant (at p<0.05) in the previous analyses

|  | **Bullying by peers** | | | |  | **Physical abuse** | | | | |
| --- | --- | --- | --- | --- | --- | --- | --- | --- | --- | --- |
| **Domains of functioning^a^** | **Flourishing / exposed to bullying by peers n (%)** | **Flourishing / exposed to other types of victimisation n (%)** | **OR (95% CI) adjusted^b^ (Ref=other types of victimisation)** | **P value** |  | **Flourishing / exposed to physical abuse n (%)** | **Flourishing / exposed to other types of victimisation n (%)** |  | **OR (95% CI) adjusted^b^ (Ref=other types of victimisation)** | **P value** |
| **Social well-being** |  |  |  |  |  |  |  |  |  |  |
| Perceived social status |  |  | - |  |  | 11 (10.5) | 82 (19.1) |  | 0.60 (0.27, 1.31) | 0.199 |
| Perceived social support | 53 (29.9) | 164 (45.1) | **0.52 (0.34, 0.79)** | **0.002** |  |  |  |  | - |  |
| **Education and cognition** |  |  |  |  |  |  |  |  |  |  |
| Perception of ability to get ahead in life | 45 (25.1) | 120 (33.0) | 0.68 (0.45, 1.03) | 0.069 |  |  |  |  | - |  |
| Spatial Span Reversed | 42 (23.7) | 76 (21.0) | 1.28 (0.81, 2.01) | 0.295 |  |  |  |  | - |  |
| **Mental well-being** |  |  |  |  |  |  |  |  |  |  |
| Life satisfaction |  |  | - |  |  |  |  |  | - |  |
| ^a^ Each domain of functioning was tested in separate binary logistic regression models; ^b^ Adjusted for biological sex at birth, family socioeconomic status at age 5, family history of psychiatric disorders assessed at age 12, and intelligence quotient at age 5.  CI, confidence interval; OR, odds ratio.  All analyses account for the non-independence of twin observations.  Flourishing was defined using standardised residuals. As a result, analyses included only individuals with complete data for each regression, and sample sizes therefore varied across outcomes.  Bold text indicates statistically significant result (p < 0.05).  Victimised individuals with standardised residuals in the top 25th percentile were considered to have enhanced flourishing. | | | | | | | | | | |

**Supplementary Table 12 (continued)**

|  | **Sexual abuse** | | | |  | **Emotional abuse and neglect** | | | | |
| --- | --- | --- | --- | --- | --- | --- | --- | --- | --- | --- |
| **Domains of functioning^a^** | **Flourishing / exposed to sexual abuse n (%)** | **Flourishing / exposed to other types of victimisation n (%)** | **OR (95% CI) adjusted^b^ (Ref=other types of victimisation)** | **P value** |  | **Flourishing / exposed to emotional abuse and neglect n (%)** | **Flourishing / exposed to other types of victimisation n (%)** |  | **OR (95% CI) adjusted^b^ (Ref=other types of victimisation)** | **P value** |
| **Social well-being** |  |  |  |  |  |  |  |  |  |  |
| Perceived social status |  |  | - |  |  | 2 (3.4) | 91 (19.1) |  | **0.21 (0.05, 0.99)** | **0.048** |
| Perceived social support |  |  | - |  |  |  |  |  | - |  |
| **Education and cognition** |  |  |  |  |  |  |  |  |  |  |
| Perception of ability to get ahead in life |  |  | - |  |  |  |  |  | - |  |
| Spatial Span Reversed | 1 (7.1) | 117 (22.3) | 0.27 (0.04, 1.79) | 0.173 |  |  |  |  | - |  |
| **Mental well-being** |  |  |  |  |  |  |  |  |  |  |
| Life satisfaction | 1 (6.7) | 147 (28.0) | 0.20 (0.02, 1.70) | 0.142 |  |  |  |  | - |  |
| ^a^ Each domain of functioning was tested in separate binary logistic regression models; ^b^ Adjusted for biological sex at birth, family socioeconomic status at age 5, family history of psychiatric disorders assessed at age 12, and intelligence quotient at age 5.  CI, confidence interval; OR, odds ratio.  All analyses account for the non-independence of twin observations.  Flourishing was defined using standardised residuals. As a result, analyses included only individuals with complete data for each regression, and sample sizes therefore varied across outcomes.  Bold text indicates statistically significant result (p < 0.05).  Victimised individuals with standardised residuals in the top 25th percentile were considered to have enhanced flourishing. | | | | | | | | | | |

**Supplementary Table 13.** Associations between exposure to poly-victimisation and flourishing in the different domains of functioning using an enhanced definition of flourishing

| **Domains of functioning^a^** | **Flourishing / exposed to poly-victimisation n (%)** | **Flourishing / exposed to single victimisation n (%)** | **OR (95% CI) adjusted^b^** | **P value** |
| --- | --- | --- | --- | --- |
| **Social well-being** |  |  |  |  |
| Perceived social status | 10 (7.9) | 83 (20.3) | 0.48 (0.21, 1.11) | 0.085 |
| **Education and cognition** |  |  |  |  |
| Perception of ability to get ahead in life | 37 (28.7) | 128 (30.9) | 0.83 (0.51, 1.34) | 0.440 |
| **Physical health** |  |  |  |  |
| Pace of biological ageing | 15 (13.8) | 96 (27.8) | **0.41 (0.22, 0.79)** | **0.007** |
| **Mental well-being** |  |  |  |  |
| Life satisfaction | 328 (21.9) | 120 (29.1) | 0.63 (0.37-1.09) | 0.099 |
| ^a^ Each domain of functioning was tested in separate binary logistic regression models; ^b^ Adjusted for biological sex at birth, family socioeconomic status at age 5, family history of psychiatric disorders assessed at age 12, and intelligence quotient at age 5.  CI, confidence interval; OR, odds ratio.  All analyses account for the non-independence of twin observations.  Flourishing was defined using standardised residuals. As a result, analyses included only individuals with complete data for each regression, and sample sizes therefore varied across outcomes.  Bold text indicates statistically significant result (p < 0.05).  Victimised individuals with standardised residuals in the top 25th percentile were considered to have enhanced flourishing. | | | | |

**Supplementary references**

1 Trouton A, Spinath FM, Plomin R. Twins early development study (TEDS): a multivariate, longitudinal genetic investigation of language, cognition and behavior problems in childhood. *Twin Res* 2002; **5**: 444–8.

2 Moffitt and E‐Risk Study Team. Teen-aged mothers in contemporary Britain. *J Child Psychol Psychiatry* 2002; **43**: 727–42.

3 CACI Information Services. ACORN user guide. London, UK: CACI; 2006.

4 Danese A, Moffitt TE, Arseneault L*, et al.* The origins of cognitive deficits in victimized children: implications for neuroscientists and clinicians. *Am J Psychiatry* 2017; **174**: 349–61.

5 Straus MA, Gelles RJ, Asplund LM. Measuring intrafamily conflict and violence: The Conflict Tactics (CT) scales. In: Ed. MA Straus and RG Gelles, editor. Physical violence in American families: Risk factors and adaptations to violence in 8,145 families. New Brunswick: Transaction Press; 1990. p. 403–24.

6 Magdol L, Moffitt TE, Caspi A*, et al.* Developmental antecedents of partner abuse: a prospective-longitudinal study. *J Abnorm Psychol* 1998; **107**: 375.

7 Shakoor S, Jaffee SR, Andreou P*, et al.* Mothers and children as informants of bullying victimization: results from an epidemiological cohort of children. *J Abnorm Child Psychol* 2011; **39**: 379–87.

8 Bowes L, Maughan B, Ball H*, et al.* Chronic bullying victimization across school transitions: the role of genetic and environmental influences. *Dev Psychopathol* 2013; **25**: 333–46.

9 Dodge KA, Bates JE, Pettit GS. Mechanisms in the cycle of violence. *Science* 1990; **250**: 1678–83.

10 Lansford JE, Dodge KA, Pettit GS*, et al.* A 12-year prospective study of the long-term effects of early child physical maltreatment on psychological, behavioral, and academic problems in adolescence. *Arch Pediatr Adolesc Med* 2002; **156**: 824–30.

11 Finkelhor D, Ormrod RK, Turner HA. Poly-victimization: a neglected component in child victimization. *Child Abuse Negl* 2007; **31**: 7–26.

12 Bernstein DP, Fink L. Manual for the childhood trauma questionnaire. New York: Psychological Corporation; 1998.

13 Fink LA, Bernstein D, Handelsman L*, et al.* Initial reliability and validity of the childhood trauma interview: a new multidimensional measure of childhood interpersonal trauma. *Am J Psychiatry* 1995; **152**: 1329–35.

14 Cambridge Cognition. CANTAB Eclipse Test Administration Guide: Cambridge Cognition; 2006.

15 Stith SM, Liu T, Davies LC*, et al.* Risk factors in child maltreatment: A meta-analytic review of the literature. *Aggression and violent behavior* 2009; **14**: 13–29.

16 Haider ZF, von Stumm S. Predicting educational and social–emotional outcomes in emerging adulthood from intelligence, personality, and socioeconomic status. *J Pers Soc Psychol* 2022; **123**: 1386.

17 Brummelhuis IA, Kop WJ, Videler AC. Psychological and physical wellbeing in adults who grew up with a mentally ill parent: A systematic mixed-studies review. *Gen Hosp Psychiatry* 2022; **79**: 162–76.

18 Wechsler D. Wechsler Preschool and Primary Scale of Intelligence-Revised. London: The Psychological Corporation, Harcourt Brace and Company; 1990.

19 Sattler JM. Assessment of Children: WISC-III and WPPSI-R supplement. San Diego: JM Sattler; 1992.

20 Trzesniewski KH, Moffitt TE, Caspi A*, et al.* Revisiting the association between reading achievement and antisocial behavior: new evidence of an environmental explanation from a twin study. *Child Dev* 2006; **77**: 72–88.

21 Weissman MM, Wickramaratne P, Adams P*, et al.* Brief screening for family psychiatric history: the family history screen. *Arch Gen Psychiatry* 2000; **57**: 675–82.

22 Milne BJ, Moffitt TE, Crump R*, et al.* How should we construct psychiatric family history scores? A comparison of alternative approaches from the Dunedin Family Health History Study. *Psychol Med* 2008; **38**: 1793–802.
